# MTCH2 regulates NRF2-mediated RRM1 expression to promote melanoma proliferation and dacarbazine insensitivity

**DOI:** 10.1038/s41419-025-07618-9

**Published:** 2025-04-09

**Authors:** Xuedan Zhang, Enjiang Li, Yingmin Kuang, Yanlong Gai, Yu Feng, Yu Huang, Zhenyan Wei, Junzi Niu, Song Yu, Zhe Yang, Qiao Zhang, Buqing Sai, Yuechun Zhu

**Affiliations:** 1 Department of Biochemistry and Molecular Biology, School of Basic Medicine, Kunming Medical University, Kunming, 650500 Yunnan China; 2 Department of Organ Transplantation, First Affiliated Hospital of Kunming Medical University, Kunming, 650500 Yunnan China; 3 Department of Pathology, First Affiliated Hospital of Kunming Medical University, Kunming, 650500 Yunnan China

**Keywords:** Melanoma, Cancer therapeutic resistance

## Abstract

Melanoma is among the 10 most prevalent malignant tumors, posing a significant threat to human health. A detailed understanding of the molecular mechanisms driving its progression is crucial for advancing treatment strategies and outcomes. Based on bioinformatic analysis and experimental validation, this study identified mitochondrial carrier homolog 2 (MTCH2) as a key regulator of melanoma proliferation. Mechanistically, MTCH2 enhanced the expression and nuclear translocation of nuclear factor (erythroid-derived-2)-like 2 (NRF2), which up-regulated ribonucleotide reductase subunit M1 (RRM1) expression, thereby promoting melanoma cell proliferation. Targeting RRM1 in combination with dacarbazine significantly inhibited tumor growth in nude mouse xenograft models. These findings elucidate a mechanistic link between MTCH2 and the NRF2-RRM1 axis in melanoma proliferation and highlight potential therapeutic targets for intervention.

## Introduction

Melanoma exhibits a high metastatic potential, a high mortality rate, and resistance to radiotherapy and chemotherapy [1]. Given these clinical challenges, elucidating the molecular mechanisms that drive melanoma progression is crucial for improving clinical efficacy. To sustain its rapid proliferation, melanoma cells maintain an expanded nucleotide pool by enhancing nucleotide biosynthesis while suppressing nucleotide degradation [2]. Ribonucleotide reductase subunit M1 (RRM1) is a key enzyme responsible for converting ribonucleotide diphosphate to deoxyribonucleotide diphosphate, a crucial step in DNA synthesis [3, 4]. In gastric cancer, RRM1 overexpression is associated with poor prognosis in advanced-stage patients [5], whereas in thyroid cancer, RRM1 promotes DNA synthesis and tumor cell proliferation, migration, and invasion [6]. To date, however, its role in melanoma has yet to be elucidated.

Dacarbazine, a first-line chemotherapeutic agent for melanoma, exerts cytotoxic effects by inducing DNA damage during replication [7]. Drug resistance, particularly to dacarbazine, is a major contributor to the low cure rates in melanoma. Studies have indicated that melanoma cells exhibit reduced sensitivity to dacarbazine in the presence of deoxyribonucleotides [8], suggesting a link between drug sensitivity and DNA metabolism. Understanding the regulatory mechanisms governing nucleotide synthesis may therefore provide insights into therapeutic vulnerabilities in melanoma.

Nuclear factor (erythroid-derived-2)-like 2 (NRF2) plays a central role in cellular redox homeostasis and transcriptional regulation. Under physiological conditions, NRF2 is primarily localized in cytoplasm, but translocates to the nucleus upon activation, where it regulates gene expression [9]. Our previous research demonstrated that NRF2 promotes melanoma progression by targeting phosphoribosyl pyrophosphate synthase 1, an enzyme that catalyzes the conversion of ribose-5-phosphate to 5-phosphoribosyl-1-phosphate, a key rate-limiting step in purine nucleotide biosynthesis [10]. NRF2 has also been implicated in nucleotide metabolism and cancer recurrence [11]. In head and neck squamous cell carcinoma, NRF2 enhances nucleotide biogenesis by up-regulating glucose-6-phosphate dehydrogenase and transketolase, thereby facilitating malignant progression [12]. However, the role of NRF2 in regulating RRM1 expression in melanoma remains unexplored.

Mitochondrial carrier homolog 2 (MTCH2), an outer mitochondrial membrane protein, is involved in apoptotic regulation [13]. MTCH2 deficiency has been shown to disrupt mitochondrial structure and function, leading to fragmentation and elevated oxidative stress [14, 15]. Abnormal MTCH2 expression has also been linked to tumorigenesis. In malignant gliomas, MTCH2 knockdown weakens tumor invasiveness and increases sensitivity to temozolomide [16]. In breast cancer, MTCH2 promotes proliferation and cell cycle progression via the PI3K/Akt pathway [17]. Furthermore, apolipoprotein C1 (APOC1) has been shown to drive osteosarcoma progression through MTCH2-dependent mechanisms [18]. However, the role of MTCH2 in melanoma remains undefined.

Based on this background, we hypothesized the following: (1) MTCH2 regulates NRF2 expression, while NRF2 transcriptionally activates RRM1 to promote melanoma proliferation; and (2) elevated RRM1 expression reduces melanoma sensitivity to dacarbazine. This study aimed to elucidate the molecular mechanisms underlying melanoma progression and provide a theoretical foundation for the development of novel therapeutic strategies.

## Materials and methods

### Drugs

ML385 (HY-100523, Med Chem Express), sulforaphane (HY-13755, Med Chem Express), and dacarbazine (HY-B0078, Med Chem Express) were dissolved in dimethylsulfoxide (DMSO, D8371, Solarbio Life Sciences) at a concentration of 10 mM.

### Cell culture

Melanoma cells were cultured in Dulbecco’s Modified Eagle Medium (DMEM) (with L-glutamine) supplemented with 10% fetal bovine serum (FBS) and 1% penicillin/streptomycin. Cells were maintained in a 37 °C incubator with 5% CO_2_, with passaging every 2 days. The melanoma cells were purchased from the Cell Bank of the Chinese Academy of Sciences.

### Cell transfection

A375 and SK-MEL-21 cells were transfected with overexpression or knockdown vectors, with CON335 and CON313 serving as corresponding controls. In brief, when cell density reached 60% confluency in a 6-well plate, the corresponding viral constructs were introduced. After 48 h, transfected cells were selected using 5 μg/mL puromycin, ensuring the elimination of non-transfected populations. Selection was maintained through serial passaging with continued puromycin exposure until over 95% of cells exhibited green fluorescent protein (GFP) fluorescence under a microscope. Successful transfection was confirmed by western blotting and quantitative polymerase chain reaction (qPCR).

### RNA preparation and quantitative real-time PCR

Cells were harvested and lysed in 1 mL of TRIzol reagent, followed by thorough mixing. After incubation on ice for 5 min, 200 µL of chloroform was added and briefly mixed. Phase separation was achieved by centrifugation at 4 °C for 10 min, with the resulting supernatant mixed with an equal volume of isopropanol. Following centrifugation at 4 °C for an additional 10 min, the supernatant was discarded, and the RNA pellet was resuspended in DEPC-treated water. The extracted RNA was stored at −20 °C until further use. cDNA synthesis was performed using a reverse transcription kit (K1622, Thermo Fisher Scientific). Gene expression levels were quantified via qPCR using SYBR Green (Roche AG, Switzerland). The primer pairs used for amplifying target genes are shown in Table 1.

**Table 1:** Primer sequences used for qPCR.

Gene	Primer
*MTCH2*	F: 5′-3′: GGTCTTGTTCCTCGCCTTCT
	R: 5′-3′: TGGTAGAAACCCCACTGTCC
*RRM1*	F:5′-3′: CGCCTGTCAGTCTGTGAGGC
	R:5′-3′: CCGTGACGCACAAAGGGGC
*U6*	F:5′-3′: CTCGCTTCGGCAGCACA
	R:5′-3′: AACGCTTCACGAATTTGCGT

### Cell proliferation assay

Cell proliferation was assessed using an MTS assay. Cells were seeded in a 96-well plate at a density of approximately 5000 cells per well. After incubation, proliferation was measured using an MTS assay kit (PF0004, Proteintech). Absorbance was recorded at 490 nm using an enzyme linked immunosorbent assay (ELISA) reader (51119200, Thermo Fisher Scientific).

### EdU assay

To evaluate DNA synthesis and cell proliferation, the EdU assay was performed. In brief, cells were seeded overnight and subsequently incubated with 1× EdU working solution for 2 h. Cells were then fixed with 4% paraformaldehyde at room temperature for 10 min, followed by permeabilization with 0.1% Triton X-100. EdU incorporation was detected using the Click-iT reaction system, with each well incubated with 100 µL of Click reaction solution for 30 min in the dark. Fluorescence imaging was performed using a Leica DM4B fluorescence microscope (×200 magnification). The assay was conducted using the BeyClick EdU reagent kit (#C0075S, Beyotime).

### Western blot analysis

Cells were harvested and lysed on ice for 30 min in RIPA lysis buffer (R0020, Solarbio Life Sciences). Lysates were centrifuged at 4 °C for 10 min, and protein concentrations were quantified using the BCA^TM^ protein detection kit (P1511, Applegen). Equal amounts of protein were separated by 8–15% sodium dodecyl-sulfate polyacrylamide gel electrophoresis (SDS-PAGE) and transferred onto polyvinylidene fluoride (PVDF) membranes (IPVH00010, Millipore). Subsequently, the membranes were blocked with 5% skim milk at room temperature for 2 h before incubation with primary antibodies overnight at 4 °C. After washing, the membranes were incubated with secondary antibodies at room temperature for 2 h. The chemiluminescence signal was amplified using an enhanced chemiluminescence (ECL) kit (K-12045-D50, Advansia), and high-quality images were obtained using the Bio Rad ChemiDoc XRS system (BioRad). β-actin was used as a reference control. All experiments were repeated at least three times. The primary antibodies used included anti-β-actin (66009-1-Ig, Proteintech), anti-MTCH2 (16888-1-AP, Proteintech), anti-RRM1 (10526-1-AP, Proteintech), anti-CDK2 (10122-1-AP, Proteintech), anti-cyclin E1 (115544-1-AP, Proteintech), and anti-NRF2 (16396-1-AP, Proteintech).

### Immunofluorescence

Cells were seeded onto glass coverslips and fixed with paraformaldehyde for 10 min. Following three phosphate-buffered saline (PBS) washes, the cells were incubated with 1% sodium citrate for 10 min and washed again with PBS three times. Permeabilization was performed using 0.1% Triton X-100 (T-8200, Solarbio Life Sciences, Beijing, China) for 10 min, followed by three additional PBS washes. Cells were then blocked with 5% goat serum at room temperature for 2 h before incubation with primary antibodies overnight at 4 °C. After three washes with PBST, the slides were incubated with secondary antibodies at 37 °C for 2 h. Nuclear staining was performed using 4′,6-diamidino-2-phenylindole (DAPI). The primary antibodies used included anti-RRM1 (10526-1-AP, Proteintech) and anti-NRF2 (16396-1-AP, Proteintech), while secondary antibodies included Alexa Fluor™ 647 and Alexa Fluor™ 488.

### Melanoma xenograft model

To establish a melanoma xenograft model, 40 male BALB/c nude mice (6 to 8 weeks old) were obtained from the Department of Experimental Animals, Kunming Medical University (China). The mice were housed in a temperature- and humidity-controlled room under a 12-h light/dark cycle, with free access to food and water. Each mouse was subcutaneously injected with 100 µL of PBS containing 1 × 10^7^ melanoma cells into one side of the armpit. Tumors were collected 20 days post-inoculation. For chemotherapy experiments, mice were randomly assigned to four groups. The control group received intraperitoneal injections of physiological saline, while the experimental group received intraperitoneal injections of 20 mg/kg dacarbazine. Tumor volume was measured every two days using a vernier caliper, and samples were harvested after 20 days. All animal experiments were approved by the Institutional Animal Care and Use Commission of Kunming Medical University (Approval number: KMMUD2025041).

### Flow cytometry

Cell cycle distribution was analyzed by flow cytometry. Cells were fixed in 95% ethanol at 4 °C for 24 h, washed with PBS, and stained with propidium iodide (PI, BD, 550825) for 15 min. Samples were then analyzed using a BD FACS Celesta™ flow cytometer.

For apoptosis assessment, 1 × 10^6^ cells were collected and stained with an Annexin V PE/7-AAD staining kit (Abcam, BA00207). The percentage of apoptotic cells was quantified by flow cytometry.

### Chromatin immunoprecipitation (ChIP) assay

ChIP assays were performed using a commercially available kit (Cambridge Abcam, USA) following the manufacturer’s protocols. Briefly, cells were harvested and crosslinked with formaldehyde at room temperature for 10 min to preserve protein-DNA interactions. The crosslinking reaction was terminated by the addition of glycine, followed by cell lysis in the presence of protease inhibitors. Chromatin was fragmented by sonication, and lysates were centrifuged at 4 °C for 10 min to collect the supernatant. A 20 µL aliquot was reserved as input control and stored at −20 °C, while the remaining lysate was evenly divided into three fractions and incubated overnight at 4 °C with antibodies against H3, IgG, and NRF2. The next day, antibody-bound chromatin complexes were captured using protein A/G magnetic beads and incubated overnight at 4 °C on a rotating mixer. On the third day, immunoprecipitated DNA was purified and centrifuged at 1400014000 *g* for 1 min at room temperature. The supernatant was collected and stored at −20 °C for subsequent analysis by qPCR. The PCR primer sequences used for target amplification are listed in Table 2.

**Table 2:** Primer sequences used for qPCR.

NRF2 binding position (Accession number: 4780)	Primer
Position 1 (1758–1768)	F: 5′-3′: CAGCGGGCTAGGTGCTCA
	R: 5′-3′: TACCATGTTTTGTGTGGGCAA
Position 2 (1861–1871)	F:5′-3′: GCGGACGGCAGTGTTTAGTAT
	R:5′-3′: CAAAACCTAACCGGCGGGAA

### Dual-luciferase reporter assay

To assess NRF2-mediated transcriptional regulation of RRM1, the RRM1 promoter sequence was cloned into a luciferase reporter plasmid. A mutant construct was generated by introducing site-specific mutations at the NRF2 binding site. The dual-luciferase reporter vector was obtained from Wuhan Genetic Engineering (China). HEK293T cells were seeded in 24-well plates and transfected with either wild-type or mutant plasmids once they reached approximately 60% confluency. After 24 h, an NRF2 agonist was added. Cells were then harvested, and luciferase activity was measured using a dual-luciferase reporter assay kit (T002, Vigorous) following the manufacturer’s protocols. Luminescence was detected using a multi-mode functional measuring instrument (CA95051, Agilent Technologies).

### Tissue microarray and immunohistochemical analysis

The tissue microarray (MME1004i) was purchased from Taibosi Biotechnology. Protein expression levels were detected by immunohistochemical analysis. Briefly, antigen retrieval was performed using 1% sodium citrate, followed by peroxidase removal with hydrogen peroxide. Permeabilization was achieved with 0.1% Triton X-100, and nonspecific binding was blocked with 5% goat serum. Tissue sections were incubated with primary antibodies overnight at 4 °C, followed by incubation with secondary antibodies at room temperature for 30 min. Protein detection was visualized using DAB staining (Biosharp, #BL732A). Samples were mounted with neutral resin, and images were acquired using a slide scanner (Teksqray, #SQS-1000). The primary antibodies used were anti-MTCH2 (16888-1-AP, Proteintech) and anti-RRM1 (10526-1-AP, Proteintech).

### Nuclear and cytoplasmic separation

Nuclear and cytoplasmic protein separation was performed using a nuclear protein extraction kit (Solarbio, EX1470). Collected cells were first resuspended in extraction solution A, mixed thoroughly, and incubated on a shaker at 4 °C for 30 min. The lysate was centrifuged, and the supernatant was collected as the cytoplasmic protein fraction. The remaining pellet was resuspended in extraction solution B, incubated under the same conditions and centrifuged to obtain the nuclear protein fraction.

### Bioinformatics analysis

Bioinformatics analysis and data mining were conducted to investigate the expression patterns and regulatory mechanisms of MTCH2, NRF2, and RRM1 in melanoma. The GEPIA database (http://gepia2.cancer-pku.cn/) was utilized to compare the expression levels of these genes between melanoma and normal tissues, providing insights into their differential expression profiles. Functional associations of RRM1 in melanoma were byexplored using the CancerSEA database (http://biocc.hrbmu.edu.cn/CancerSEA/), allowing for the assessment of its involvement in various cellular processes. To examine potential transcriptional regulation, the JASPAR database (https://jaspar.genereg.net/) was employed to predict NRF2 binding sites within the RRM1 promoter region, as well as to aid in the design of primers for chromatin immunoprecipitation (ChIP) assays. These analyses facilitated a comprehensive understanding of the roles of MTCH2, NRF2, and RRM1 in melanoma progression.

### Statistical analysis

All statistical analyses were conducted using SPSS v26.0. Experimental data were obtained from at least three independent replicates, presented as mean ± standard deviation (SD). Prior to statistical tests, homogeneity of variance across groups was evaluated. Independent sample *t*-tests were used to compare differences between two groups, while one-way analysis of variance (ANOVA) was applied for comparisons among multiple groups. Multifactor ANOVA was used to compare differences between two or more treatment factors. A *P-*value < 0.05 was considered statistically significant. Additionally, sample size estimation was performed based on a literature review using G Power v3.1.9.7 software.

## Results

### MTCH2 is up-regulated in melanoma and correlates with tumor malignancy

**Fig. 1:**
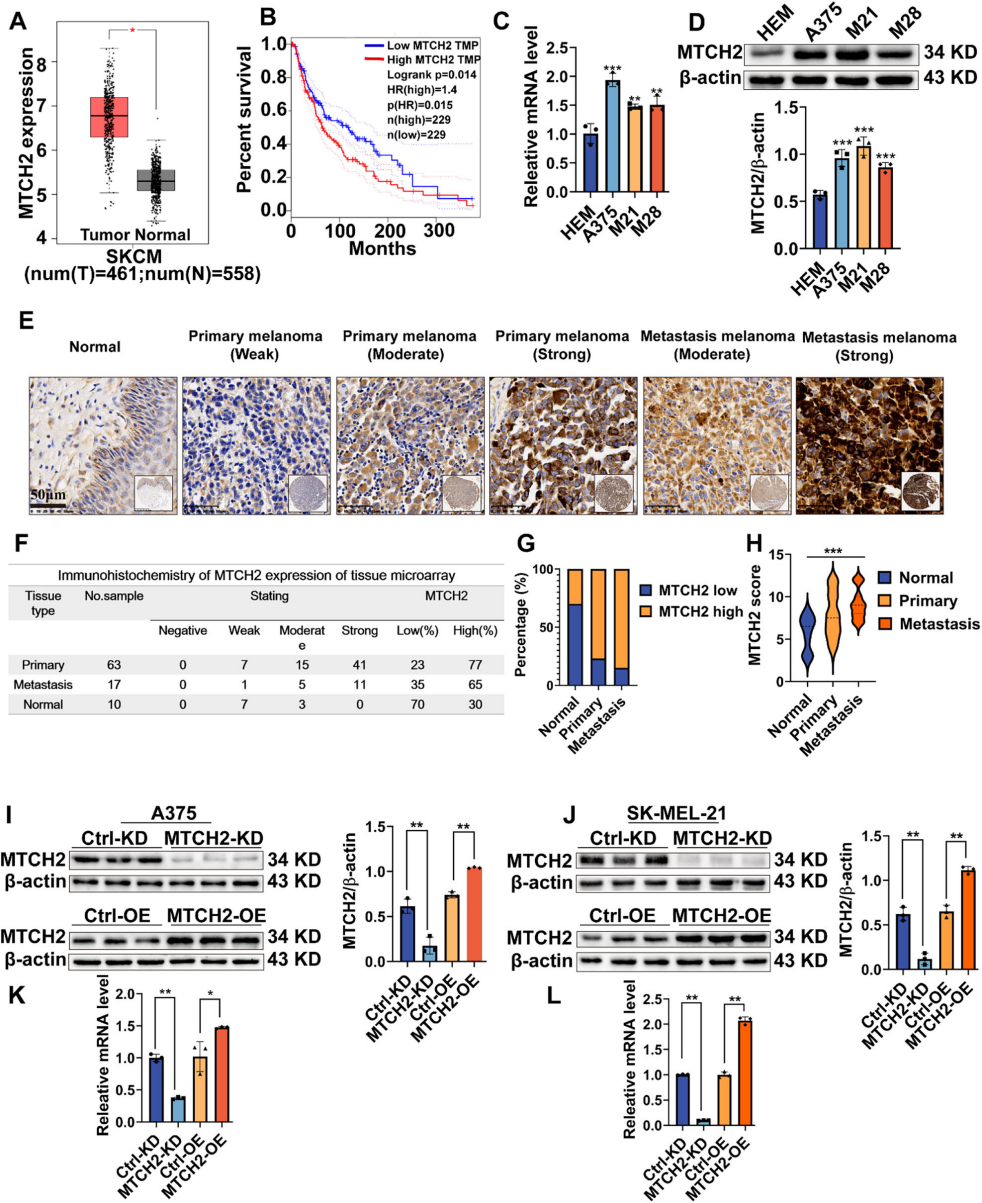
MTCH2 is highly expressed in melanoma and is related to the degree of malignancy of melanoma. **A** The expression of MTCH2 in normal tissue and melanoma tissue based on GEPIA database. **B** The relationship of MTCH2 with survival rate in melanoma based on TCGA data. **C**, **D** The mRNA and protein expression of MTCH2 in HEM cell and melanoma cells were analyzed by qPCR (**C**) and western blotting analysis (**D**). **E**–**H** Representative images of MTCH2 expression in the melanoma and the normal tissue microarray are shown. Scale bars = 50 μm (**E**). Immunohistochemical results of MTCH2 expression in melanoma tissue microarray (**F**). The percentage of high and low expression of MTCH2 in normal tissues, primary, and metastatic melanoma (**G**). MTCH2 score (**H**). **I**–**L** The stable MTCH2 knockdown and overexpression in A375 and SK-MEL-21 cells were established. The expression of MTCH2 were measured by western blotting (**I**, **J**) and qPCR (**K**, **L**) analysis. The data represent three independent experiments. The data as indicated the mean ± SD and was analyzed by Student’s *t* test. (*p < 0.05; **p < 0.01; ***p < 0.001).

To determine whether MTCH2 plays a role in melanoma progression, its expression and prognostic significance were analyzed in melanoma patients using the GEPIA database. Results demonstrated a significant up-regulation of MTCH2 in melanoma tissues compared to normal controls (Fig. 1A), with higher MTCH2 expression associated with poorer survival outcomes (Fig. 1B). Western blot and qPCR analyses further confirmed that MTCH2 expression at both the protein and mRNA levels was markedly elevated in melanoma cell lines compared to human epidermal melanocytes (HEM) (Fig. 1C, D). To extend these findings to clinical samples, a tissue microarray was employed to evaluate MTCH2 expression in normal and melanoma tissues. Representative images of immunohistochemical staining are shown in Fig. 1E. The tissue microarray included 80 melanoma cases, including 63 primary melanoma and 17 metastatic melanoma patients. The positive detection rates for MTCH2 in normal, primary melanoma, and metastatic melanoma tissues were 30%, 77%, and 65%, respectively (Fig. 1F, G). Additionally, MTCH2 expression scores were significantly higher in melanoma tissues than in normal tissues (Fig. 1H).

To further explore the functional role of MTCH2 in melanoma, stable MTCH2 knockdown (KD) and overexpression (OE) cell lines were successfully established in A375 and SK-MEL-21 cells. Changes in MTCH2 expression levels in these stably transfected cells were validated by western blotting (Fig. 1I, J, Supplementary Fig. 1A) and qPCR analyses (Fig. 1K, L, Supplementary Fig. 1A).

Overall, these findings indicate that MTCH2 is overexpressed in melanoma and correlates with tumor malignancy, providing a potential target for therapeutic intervention.

### MTCH2 promotes melanoma cell proliferation

**Fig. 2:**
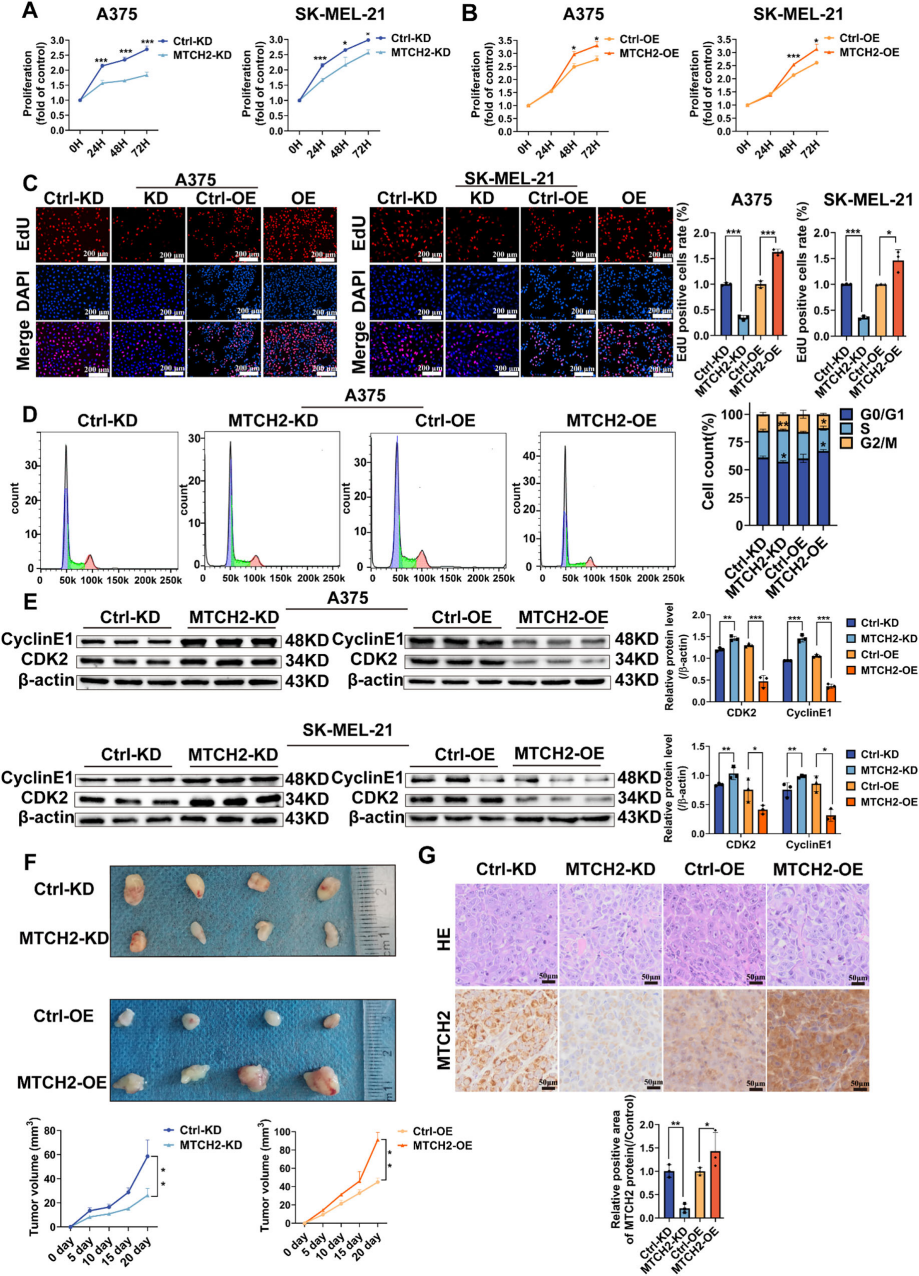
MTCH2 promotes melanoma cells proliferation in vitro and vivo. **A**, **B** The proliferation rate of MTCH2 knockdown or MTCH2 overexpressing A375, SK-MEL-21 and the corresponding control cells were detected by MTS assay. **C** EdU was used to evaluate the proliferation of MTCH2 knockdown or MTCH2 overexpressing A375 and SK-MEL-21 cells. Scale bars = 200 μm. **D** The phase distribution of MTCH2 knockdown or MTCH2 overexpressing A375 and the corresponding control cells cell cycle were detected by flow cytometry. **E** Western blot was used to detect the protein expression of Cyclin E1 and CDK2 in MTCH2 knockdown or MTCH2 overexpressing A375 and SK-MEL-21 cells. **F** BALB/c nude mice were injected with MTCH2 knockdown or MTCH2 overexpressing A375 cells and the control cells. Measure tumor volume. (*n* = 4). **G** Morphological feature of each xenograft tumor group by HE staining and IHC. Scale bars = 50 μm. The data represent three independent experiments. Each bar represents mean ± SD. *p* values were calculated using a Student’s *t* test (*p < 0.05, **p < 0.01, ***p < 0.001 vs. each control).

To further investigate the role of MTCH2 in melanoma proliferation, in vitro MTS assays and EdU staining assays were performed. The MTS assay results revealed that MTCH2 knockdown markedly reduced the proliferative capacity of A375 and SK-MEL-21 cells (Fig. 2A). Conversely, stable overexpression of MTCH2 significantly enhanced melanoma cell proliferation (Fig. 2B). Consistent with these findings, EdU staining demonstrated that MTCH2-overexpressing cells exhibited a significantly increased rate of DNA synthesis, while MTCH2-knockdown cells displayed a pronounced reduction in proliferative activity (Fig. 2C).

To further assess the impact of MTCH2 on cell cycle progression, flow cytometry was performed. Results indicated that MTCH2 knockdown led to an increase in the percentage of S phase cells and a decrease in the proportion of G0/G1 phase cells, while MTCH2 overexpression resulted in the opposite trend, with a decrease in S phase cells and an increase in G0/G1 phase cells (Fig. 2D). At the molecular level, Western blot analysis revealed that MTCH2 overexpression was associated with reduced levels of key cell cycle regulators, cyclin E1 and CDK2, while MTCH2 knockdown led to their up-regulation in A375 and SK-MEL-21 cells (Fig. 2E). These findings suggest that MTCH2 plays a pivotal role in driving melanoma cell cycle progression and proliferation.

To evaluate the effects of MTCH2 on tumor growth in vivo, A375 cells with stable MTCH2 overexpression or knockdown, alongside control cells, were subcutaneously implanted into BALB/c nude mice, and tumor growth was monitored. Results demonstrated that MTCH2 overexpression in A375 cells significantly accelerated tumor growth, whereas MTCH2 knockdown led to a marked reduction in tumorigenicity (Fig. 2F). In addition, hematoxylin and eosin (H&E) and immunohistochemical staining were performed to assess morphology and MTCH2 expression in excised tumor tissues (Fig. 2G).

Collectively, these findings demonstrate that MTCH2 promotes melanoma cell proliferation by regulating cell cycle progression and tumor growth.

### MTCH2 knockdown promotes melanoma cell apoptosis

**Fig. 3:**
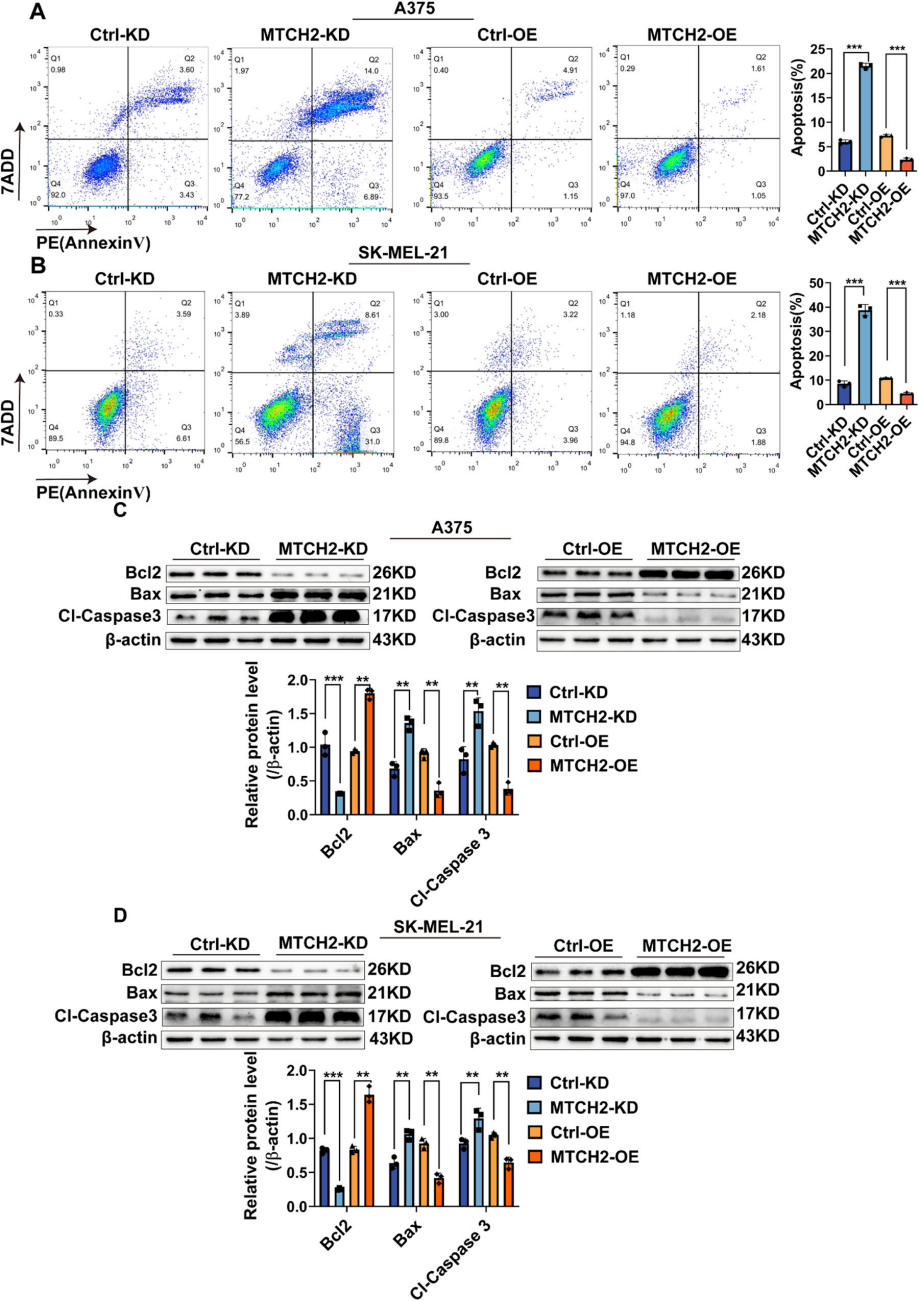
MTCH2 inhibits apoptosis in melanoma. **A**, **B** Anti-apoptotic ability of MTCH2 knockdown or MTCH2 overexpressing A375, SK-MEL-21 and the control cells were evaluated by flow cytometry. **C**, **D** The expressions of apoptosis related factors Bcl2, Bax and Cl-Caspase3 in MTCH2 knockdown or MTCH2 overexpressing A375 and SK-MEL-21. The data represent three independent experiments. Each bar represents mean ± SD. *p* values were calculated using a Student’s *t* test (*p < 0.05, **p < 0.01, ***p < 0.001 vs. each control).

To further elucidate the role of MTCH2 in melanoma, its impact on cell apoptosis was assessed via flow cytometry. Results indicated that MTCH2 knockdown significantly promoted apoptosis, while MTCH2 overexpression reduced apoptotic rates (Fig. 3A, B). Further analysis revealed that MTCH2 knockdown led to a substantial up-regulation of the pro-apoptotic factors Bax and cleaved Caspase-3 (Cl-Caspase-3), as well as a marked down-regulation in the anti-apoptotic protein Bcl-2. Conversely, MTCH2 overexpression resulted in the opposite trend (Fig. 3C, D).

Overall, these results indicate that MTCH2 inhibits melanoma cell apoptosis, thereby contributing to tumor survival and progression.

### MTCH2 regulates NRF2 and RRM1 expression

**Fig. 4:**
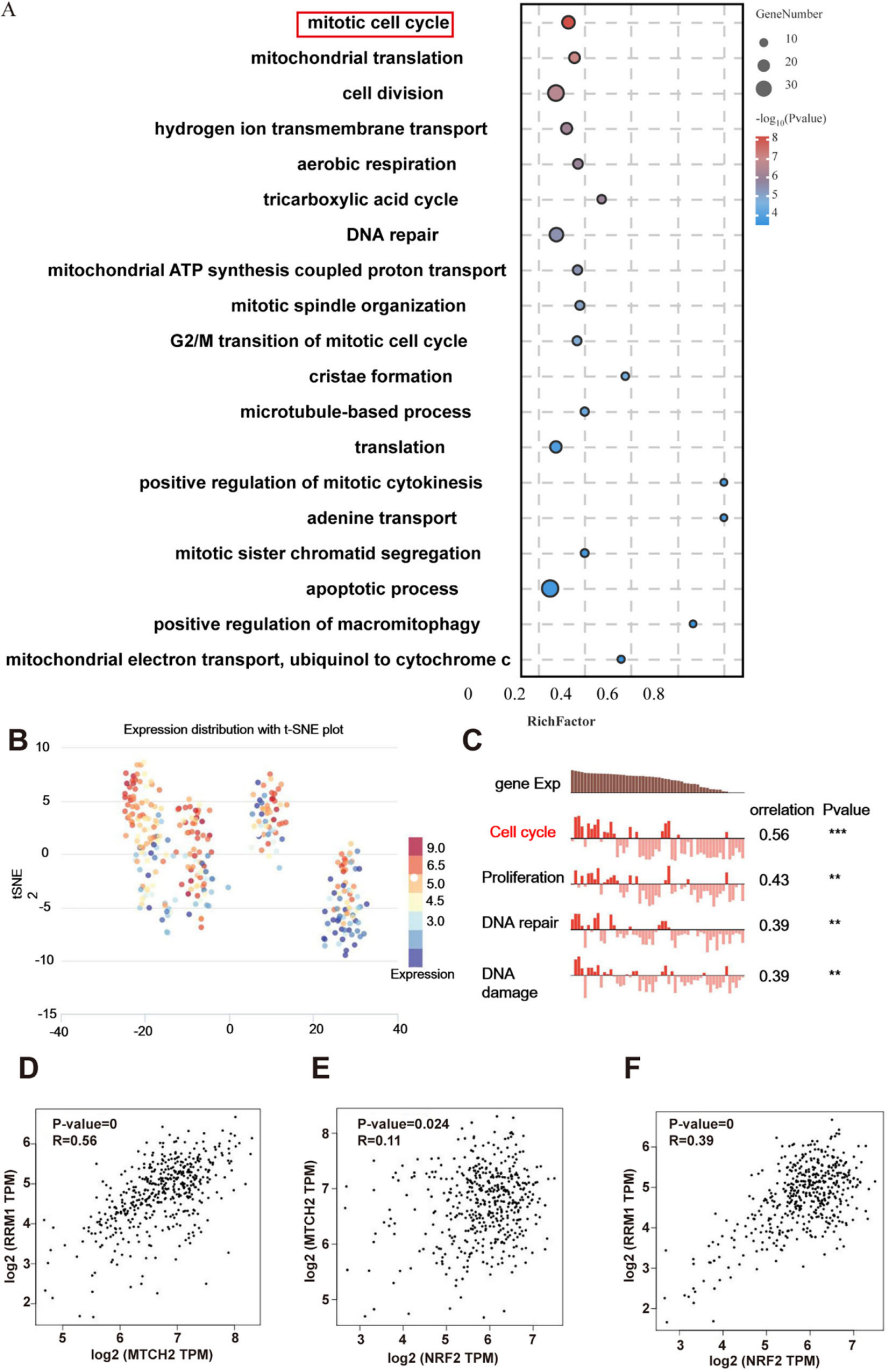
MTCH2 is positively correlated with NRF2 and RRM1 expression in melanoma. **A** Bioinformatics was used to detect the enrichment of MTCH2 in various cell processes. **B**, **C** Correlation of RRM1 with the function of 307 single cells from 3 melanoma cell lines were analyzed by *t*-SNE downscaled visualization. **D**–**F** Spearman correlation analysis of MTCH2 or RRM1 and NRF2.

**Fig. 5:**
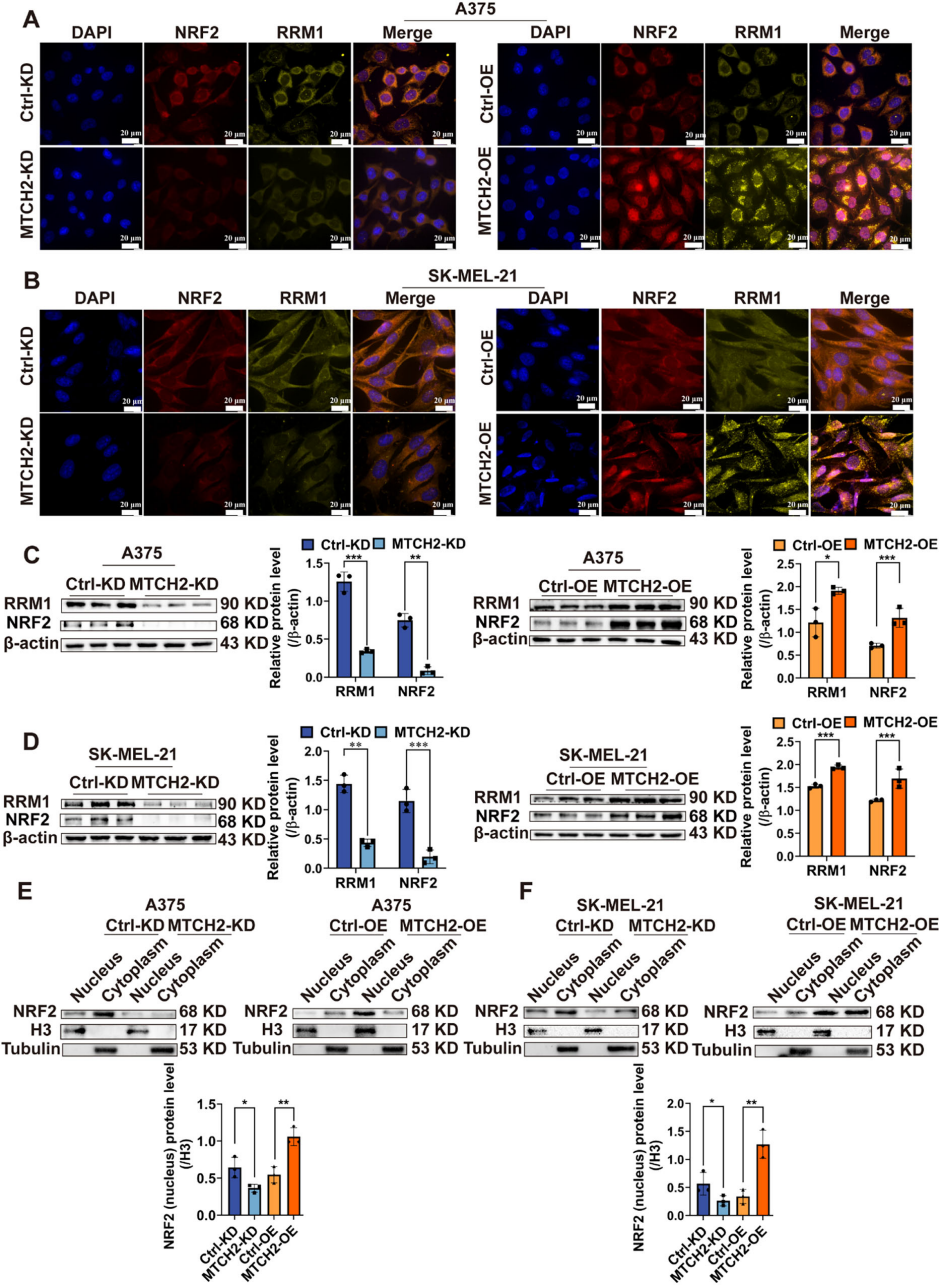
MTCH2 regulates the expression of RRM1 and NRF2. **A**, **B** Immunofluorescence analysis was used to analyze the expression of NRF2 and RRM1 in MTCH2 knockdown or MTCH2 overexpressing A375, SK-MEL-21 and the control cells. Scale bars = 20 μm. **C**, **D** The protein level of RRM1 and NRF2 were analyzed in the stably MTCH2 knockdown or MTCH2 overexpressing A375, SK-MEL-21 and that the control cells. **E**, **F** NRF2 expression in nucleus and cytoplasm of MTCH2 knockdown or MTCH2 overexpressing A375 and SK-MEL-21 cells. The data represent three independent experiments. Each bar represents mean ± SD. *p* values were calculated using a Student’s *t* test (*p < 0.05, **p < 0.01, ***p < 0.001 vs. each control).

To further explore the mechanisms by which MTCH2 regulates melanoma progression, KEGG pathway enrichment analysis was performed. Results revealed that MTCH2 was primarily enriched in the cell cycle (Fig. 4A). Additionally, single-cell transcriptomic analysis of data from the CancerSEA database was used to explore the functional relevance of RRM1 in melanoma. Notably, t-SNE visualization demonstrated that RRM1 was associated with cell cycle regulation, proliferation, and DNA damage repair (Fig. 4B, C), suggesting a potential regulatory relationship between MTCH2 and RRM1. To validate this hypothesis, GEPIA database analysis was conducted, revealing a significant positive correlation between the transcriptional expression of MTCH2 and RRM1 (Fig. 4D). Further analysis confirmed positive correlations between RRM1 and NRF2, as well as between NRF2 and MTCH2 (Fig. 4E, F). To experimentally confirm these relationships, immunofluorescence staining (Fig. 5A, B) and Western blot analysis (Fig. 5C, D) were performed to assess NRF2 and RRM1 expression in MTCH2-overexpressing and knockdown melanoma cells. Results indicated that MTCH2 overexpression led to significant up-regulation of NRF2 and RRM1 protein levels, where MTCH2 knockdown resulted in their substantial down-regulation. Additionally, Western blot analysis of nuclear and cytoplasmic fractions revealed that NRF2 was primarily localized in the nucleus of MTCH2-overexpressing A375 and SK-MEL-21 cells, while whereas the opposite trend was observed in MTCH2-knockdown cells (Fig. 5E, F).

Collectively, these findings suggest that MTCH2 promotes melanoma proliferation by regulating the NRF2 and RRM1 signaling axis, providing a mechanistic link between MTCH2 expression and melanoma progression.

### NRF2 targets and regulates RRM1 transcription

**Fig. 6:**
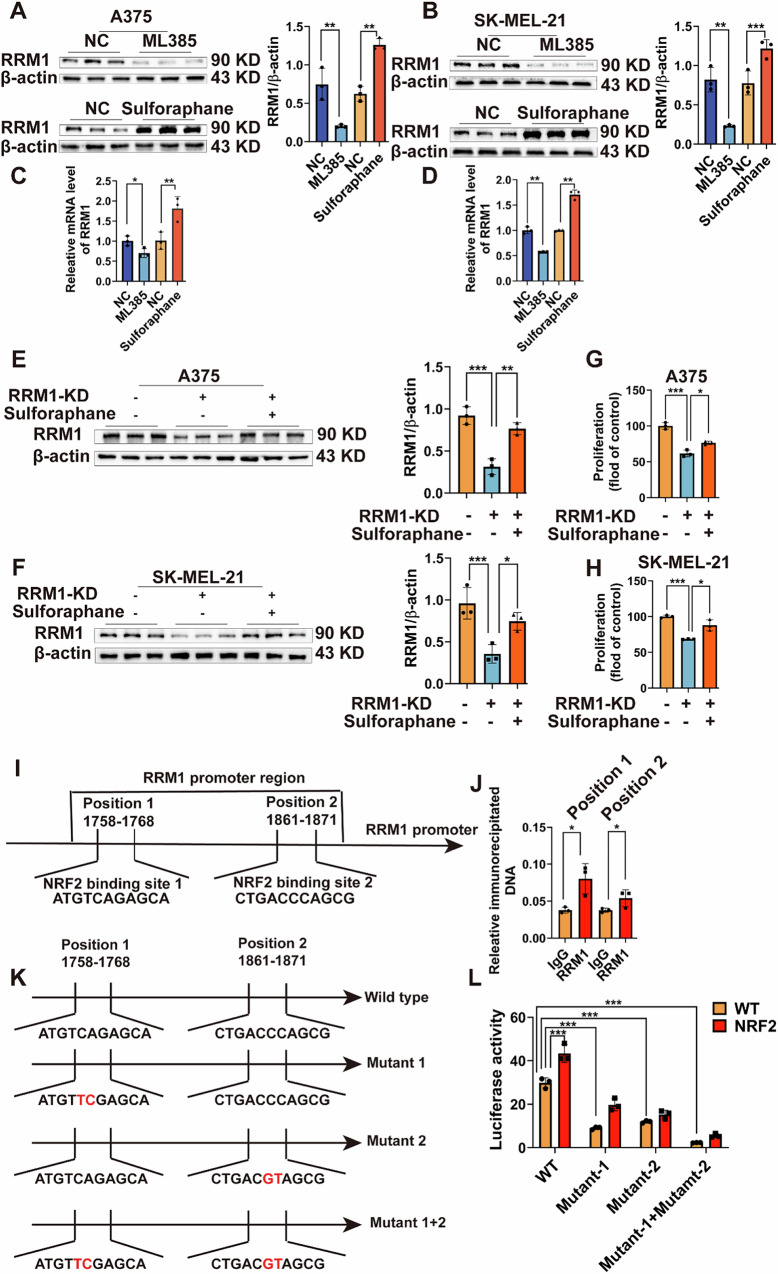
NRF2 is a transcription factor of RRM1-mediated malignant progression of melanoma. **A**–**D** Western blot and qPCR were used to detect the RRM1 expression levels in A375 and SK-MEL-21 cells treated with ML385 (NRF2 inhibitor) and Sulforaphane (NRF2 activator) for 24 h, then compared with the RRM1 protein (**A**, **B**) and mRNA levels in control cells (**C**, **D**). **E**, **F** Rescue to verify the interaction between RRM1 and NRF2 in A375 and SK-MEL-21 cells by using Western blot. **G**, **H** The cell proliferation rate was detected by MTS assay. **I** Consensus binding sites for NRF2 on the RRM1 promoter was analyzed by JASPAR database. **J** ChIP assay using NRF2 antibody and quantification of the enrichment of NRF2 binding to RRM1 promoter in A375 cells. **K** Design of luciferase reporter vector containing RRM1 promoter with a NRF2 binding site or a mutant NRF2 binding site. The red indicates mutant bases. **L** Luciferase reporter assay of NRF2 binding to RRM1 promoter. (The NRF2 group is treated with agonists). The data represent three independent experiments. Each bar represents mean ± SD. *p* values were calculated using a Student’s *t* test and multivariate analysis Of Variance (*p < 0.05, **p < 0.01, ***p < 0.001 vs. each control).

To validate the regulatory relationship between NRF2 and RRM1, A375 and SK-MEL-21 melanoma cells were treated with sulforaphane (an NRF2 activator) and ML385 (an NRF2 inhibitor). Results confirmed that NRF2 inhibition significantly reduced both RRM1 protein (Fig. 6A, B) and mRNA levels (Fig. 6C, D), whereas NRF2 activation led to a marked increase in RRM1 expression. Additionally, rescue experiments demonstrated that RRM1 knockdown (Supplementary Fig. 1B) significantly reduced melanoma cell proliferation and RRM1 protein levels. However, treatment of RRM1-deficient cells with sulforaphane partially restored both RRM1 expression (Fig. 6E, F) and proliferation capacity (Fig. 6G, H). These findings indicate that NRF2 plays a crucial role in regulating RRM1 expression and melanoma cell proliferation.

To elucidate the mechanism by which NRF2 regulates RRM1, we analyzed the JASPAR database and identified two putative NRF2-binding sites within the RRM1 promoter region (Fig. 6I). ChIP-qPCR analysis further confirmed that NRF2 was recruited to both binding site 1 (positions 1758–1768) and binding site 2 (positions 1861–1871) of the RRM1 promoter (Fig. 6J), providing direct evidence of NRF2 binding to RRM1 regulatory elements. Further validation was obtained through dual-luciferase reporter assays, which revealed that mutation of these NRF2 binding sites significantly reduced luciferase activity in plasmid-transfected HEK293T cells, confirming the functional relevance of NRF2-mediated transcriptional regulation of RRM1 (Fig. 6K, L).

Together, these results establish that NRF2 directly binds to and regulates the transcriptional expression of RRM1, thereby influencing melanoma cell proliferation.

### RRM1 expression is associated with the anti-tumor activity of dacarbazine

**Fig. 7:**
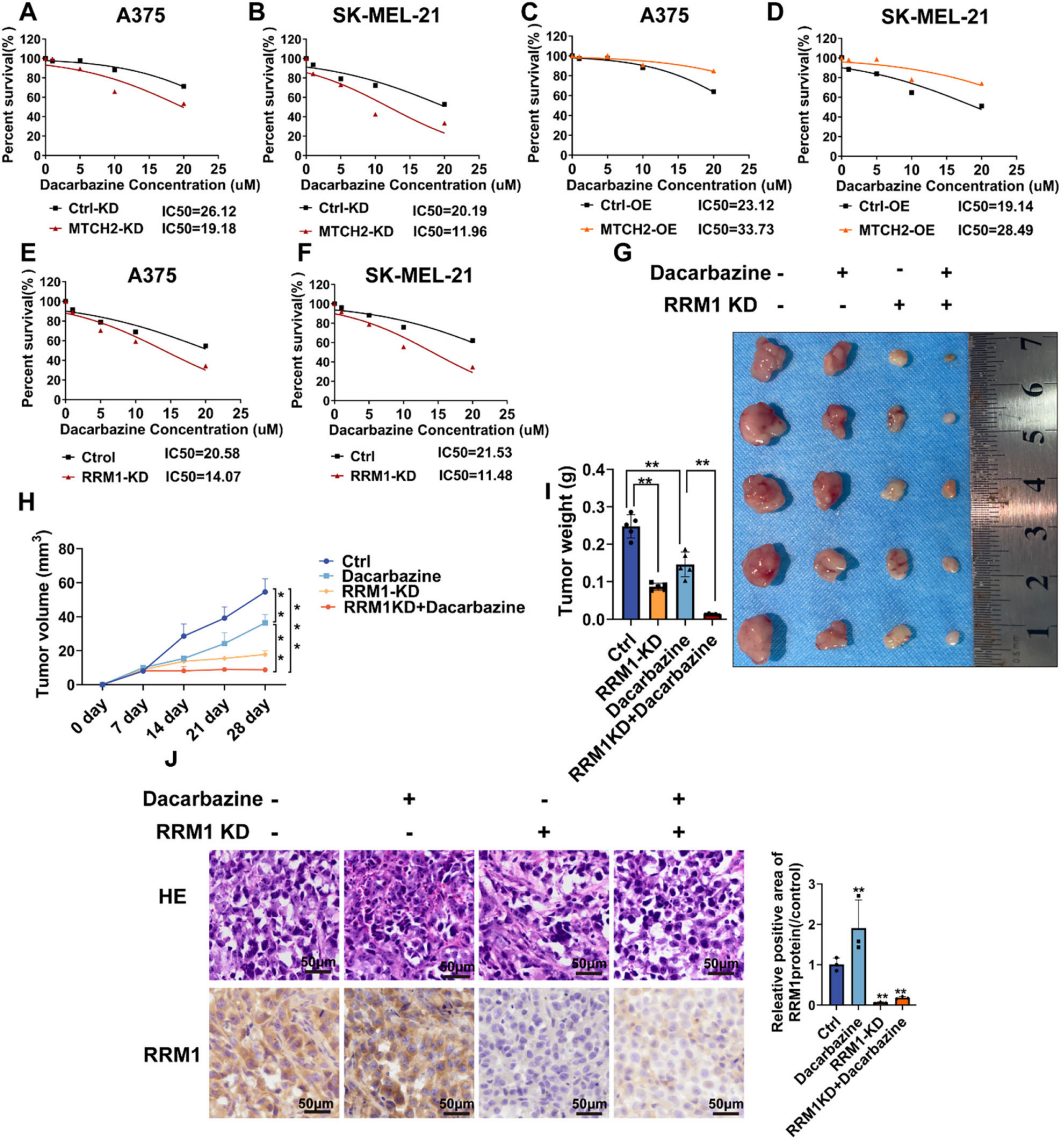
Targeting RRM1 promoted the antitumor activity of dacarbazine in vitro and in vivo. **A**–**F** Relative cell viability of A375 and SK-MEL-21 cells with MTCH2 knockdown (**A**, **B**), MTCH2 overexpressing (**C**, **D**) and RRM1 knockdown (**E**, **F**) treated with different concentrations of dacarbazine for 24 h. **G** BALB/c nude mice were injected with A375 cell that were stably transfected with RRM1 knockdown and the control. After one week of tumor injection, start treatment with dacarbazine. Representative images of mice with RRM1 control and knockdown xenograft tumors. The tumor volume (**H**) and the tumor weight (**I**) were measured. (*n* = 5/group). **J** Morphological feature of each xenograft tumor group by HE staining and IHC. The data represent three independent experiments. Scale bars = 50 μm. The data represent three independent experiments. The data related to tumor volume and weight, IHC were statistically analyzed by two-way ANOVA (*p < 0.05, **p < 0.01, ***p < 0.001).

Dacarbazine, a non-specific alkylating chemotherapeutic agent, is widely used in melanoma treatment, primarily exerting its cytotoxic effects by disrupting DNA synthesis and replication in cancer cells [19]. To determine whether RRM1 expression influences melanoma sensitivity to dacarbazine, a melanoma cell line with stable RRM1 knockdown was generated (Supplementary Fig. 1B). MTS assays were then performed to assess cell viability at various dacarbazine concentrations. The half-maximal inhibitory concentration (IC50) was determined for melanoma cells with stable MTCH2 knockdown (Fig. 7A, B), MTCH2 overexpression (Fig. 7C, D), and RRM1 knockdown (Fig. 7E, F). The results revealed that RRM1 or MTCH2 knockdown enhanced melanoma sensitivity to dacarbazine, whereas MTCH2 overexpression reduced drug susceptibility.

To further investigate whether low RRM1 expression impacts melanoma proliferation and dacarbazine sensitivity in vivo, A375 cells with stable RRM1 knockdown were subcutaneously implanted into BALB/c nude mice, and tumor growth was monitored. Results indicated that tumors formed by RRM1-knockdown cells were notably smaller and lighter than controls, indicating a suppression of tumorigenic potential. Moreover, combining dacarbazine treatment with RRM1 knockdown further reduced tumor growth rates and tumor sizes compared to dacarbazine treatment alone (Fig. 7G–I). In addition, H&E and immunohistochemical staining were performed to evaluate cell morphology and RRM1 expression levels in excised tissues (Fig. 7J).

These findings indicate that RRM1 plays a critical role in regulating melanoma cell sensitivity to dacarbazine, highlighting RRM1 as a potential therapeutic target to enhance the efficacy of this chemotherapeutic agent.

## Discussion

**Fig. 8:**
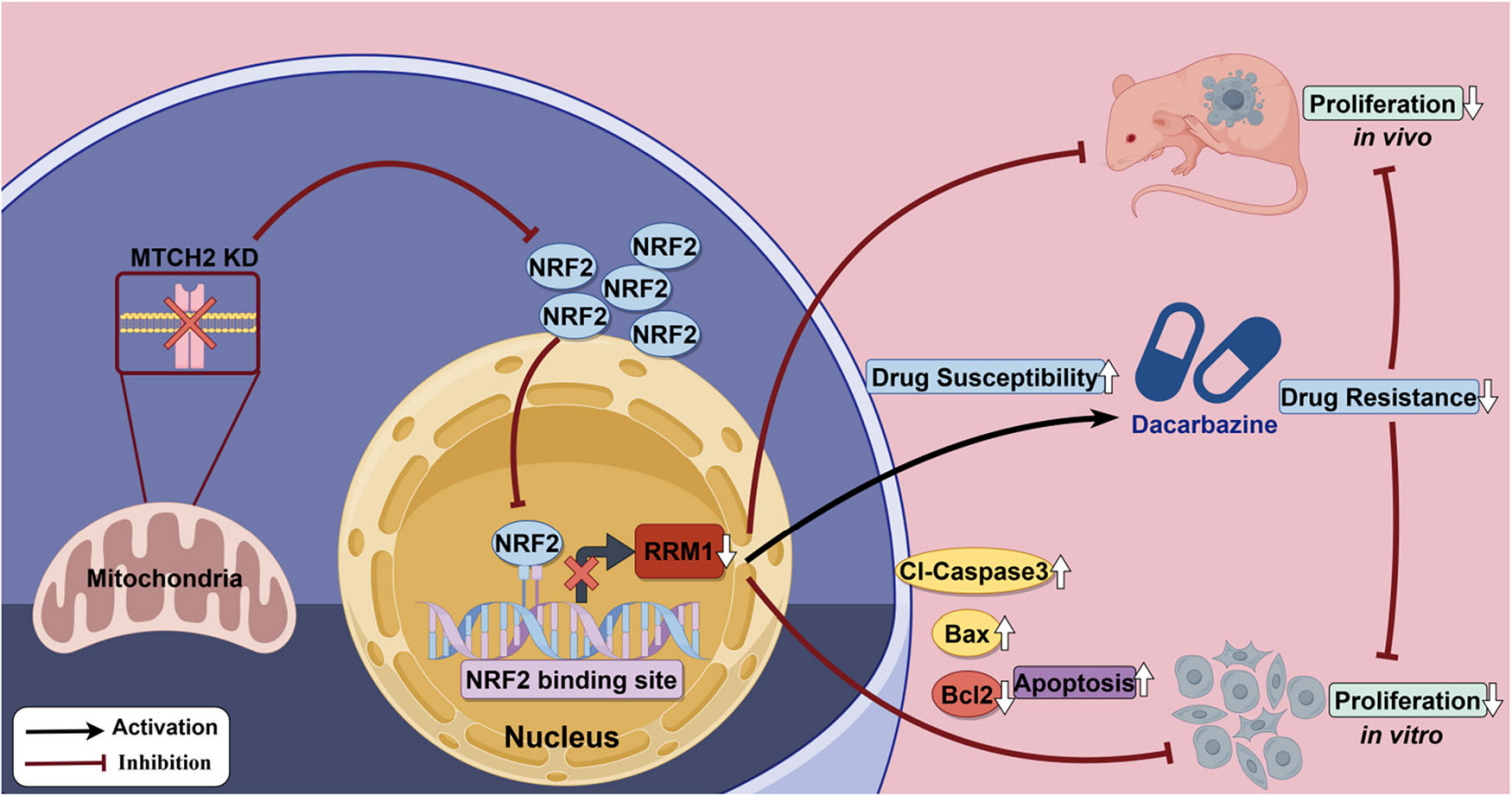
Mechanism summary diagram. When MTCH2 expression is defective (MTCH2 knockdown), the amount of NRF2 entering the nucleus is reduced, leading to decreased RRM1 transcription. Consequently, the sensitivity of melanoma cells to Dacarbazine is enhanced, effectively inhibiting their proliferation.

Our study demonstrated that elevated MTCH2 expression promoted melanoma proliferation by up-regulating NRF2 expression and nuclear translocation. NRF2, in turn, directly targeted RRM1 transcription, ultimately driving tumor cell proliferation. Notably, increased RRM1 levels reduced melanoma sensitivity to dacarbazine, highlighting a potential mechanism of chemoresistance (Fig. 8).

RRM1 is a key enzyme in deoxyribonucleotide biosynthesis, catalyzing the conversion of ribonucleoside diphosphate into deoxyribonucleoside diphosphate, a crucial step in generating deoxyribonucleoside triphosphates (dNTPs) for DNA synthesis [20]. The high metabolic demand of rapidly proliferating tumor cells necessitates increased dNTP production, making RRM1 a critical factor in tumor progression. Prior studies have shown that RRM1 knockdown significantly suppresses bladder cancer growth [4], while inhibiting RRM1 in non-small cell lung cancer reduces cell viability and induces apoptosis [21]. Similarly, RRM1 up-regulation in colorectal cancer has been linked to enhanced tumor progression [22]. Consistent with these findings, our study showed that RRM1 promoted melanoma proliferation, while its inhibition reduced tumor cell viability. Beyond its role in DNA synthesis, RRM1 is also involved in DNA damage and repair, accumulating at DNA damage sites and facilitating dNTP supply [23]. In pancreatic cancer, gemcitabine-induced DNA damage triggers RRM1 up-regulation [24], suggesting that RRM1 not only participates directly in DNA repair but also indirectly supports repair processes by maintaining nucleotide pools. Given that dacarbazine exerts its antitumor effects by inducing DNA damage [7], the rapid restoration of damaged DNA via RRM1-mediated dNTP synthesis may underlie melanoma insensitivity to dacarbazine [25]. Furthermore, previous studies have demonstrated that increased intracellular deoxyribonucleotide levels contribute to dacarbazine resistance, further supporting the inverse relationship between RRM1 expression and chemotherapy efficacy. Our findings reinforce this notion, as reducing RRM1 expression significantly enhanced dacarbazine sensitivity, whereas high RRM1 levels conferred resistance.

NRF2 is widely recognized as a key driver of tumor development [26]. In cervical cancer, NRF2 promotes metastasis by modulating epithelial-mesenchymal transition (EMT)-related factors [27], while in breast cancer, it enhances proliferation and metastasis through activation of the RhoA/ROCK signaling pathway [28]. In non-small cell lung cancer, NRF2 facilitates tumor progression by inducing autophagy [29]. In melanoma, it has been shown to support tumor growth and lung metastasis [30] and promote invasive phenotypes in BRAF-mutant melanoma cells [31]. Our study showed that NRF2 directly regulated RRM1 transcription, revealing a previously uncharacterized mechanism by which NRF2 drives melanoma proliferation. These findings provide new evidence for the role of NRF2 in nucleotide metabolism regulation, further implicating it in melanoma progression.

MTCH2, a mitochondrial outer membrane carrier protein, plays a pivotal role in apoptosis regulation by influencing mitochondrial permeability [32]. Studies have shown that MTCH2 deficiency in hematopoietic stem cells (HSCs) enhances mitochondrial oxidative phosphorylation (OXPHOS) [33], while MTCH2 knockdown in glioma cells promotes OXPHOS-induced oxidative damage [16]. These findings suggest that MTCH2 is a critical regulator of OXPHOS, which may underpin its effects on NRF2 expression and nuclear translocation. MTCH2 has been shown to promote tumor progression in multiple cancers, including glioma, osteosarcoma, and breast cancer [16–18]. Our study extended these findings to melanoma, demonstrating that MTCH2 overexpression promoted cell proliferation and altered cell cycle distribution by up-regulating cyclin E1 and CDK2 expression. Concurrently, MTCH2 inhibited cell apoptosis, up-regulated anti-apoptotic factor, and down-regulated pro-apoptotic factors Bax and Cl-Caspase-3 via the NRF2-RRM1 axis. Future studies should explore how MTCH2 influences oxidative phosphorylation in melanoma, which could provide deeper insights into its role in tumor metabolism and therapeutic resistance.

## Conclusions

This study identified MTCH2 as a key oncogenic driver in melanoma, demonstrating its role in enhancing proliferation while inhibiting apoptosis. These findings establish a foundation for targeting MTCH2 as a potential therapeutic strategy to inhibit melanoma progression. Mechanistic analysis revealed that MTCH2 regulates NRF2, which, in turn, transcriptionally activates RRM1, driving melanoma growth. Notably, aberrant RRM1 expression was shown to contribute to melanoma insensitivity to dacarbazine, offering critical insights into the mechanisms underlying chemotherapy insensitivity. These findings not only enhance the understanding of melanoma pathogenesis but also provide a theoretical basis for optimizing clinical treatment strategies. Our study highlights RRM1 as a potential predictive biomarker for dacarbazine sensitivity, paving the way for personalized therapeutic strategies in melanoma management.

## Supplementary information

Supplementary Figure1

WB original figure

## Data Availability

The original contributions presented in the study are included in the article and supplementary material. Further inquiries can be directed to the corresponding authors.
