# Supplementary material for: MTCH2 regulates NRF2-mediated RRM1 expression to promote melanoma proliferation and dacarbazine insensitivity
Source: Cell Death Dis. 2025 Apr 9;16(1):268. doi: 10.1038/s41419-025-07618-9 (PMC11982210; doi:10.1038/s41419-025-07618-9)
Supplement: Supplementary file 1 — Supplementary Figure1 [file 41419_2025_7618_MOESM1_ESM.docx]

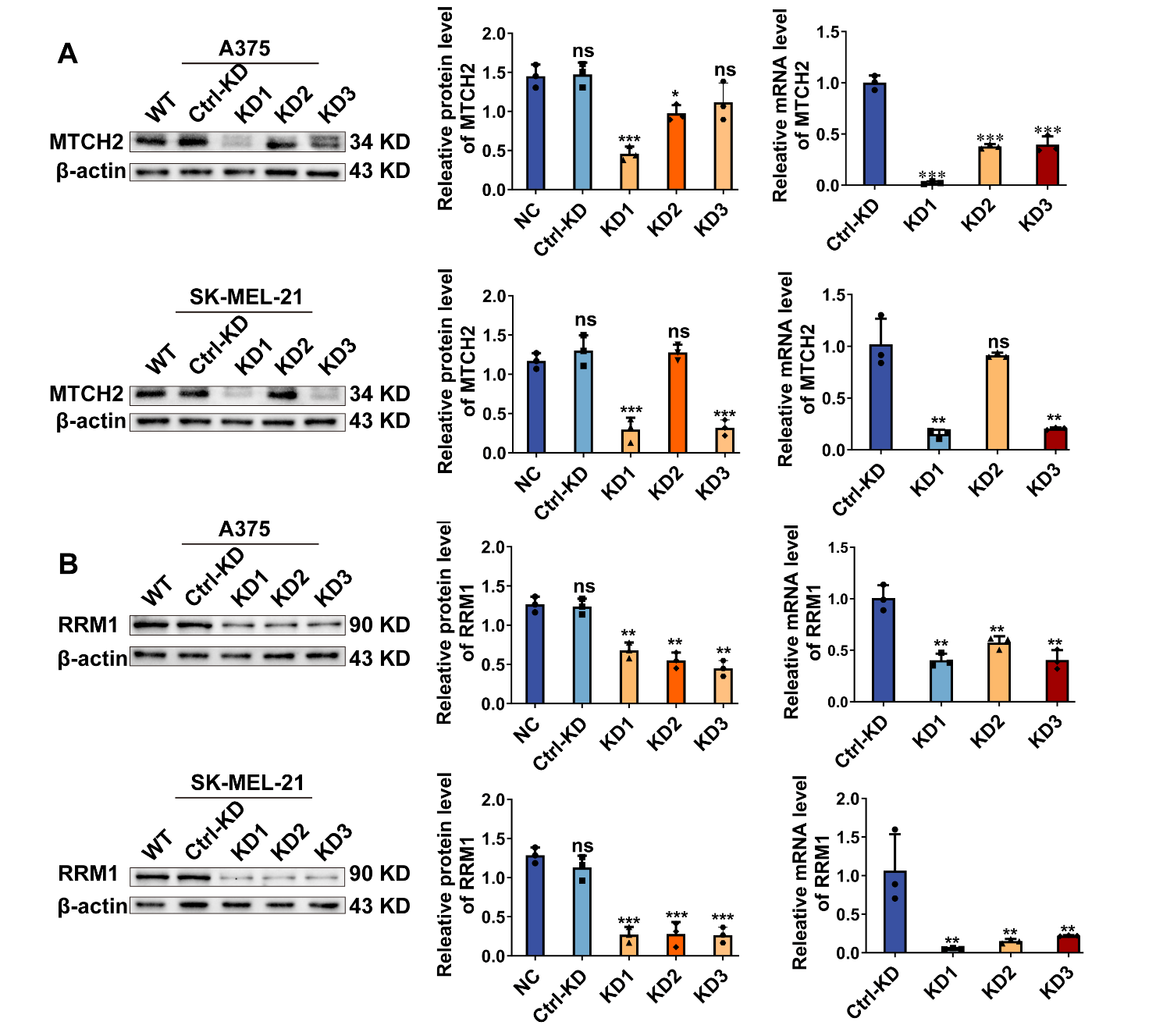


**Supplementary Figure1 Identification results of MTCH2 and RRM1 knockdown cell lines.**

(A) Construction of stably transfected cells with MTCH2 knockdown in A375 and SK-MEL-M21. Protein expression levels were detected by Western blot and mRNA expression levels were detected by real-time PCR

(B) Construction of stably transfected cells with RRM1 knockdown in A375 and SK-MEL-M21. Protein expression levels were detected by Western blot and mRNA expression levels were detected by real-time PCR

The data represent three independent experiments. Data are expressed as the mean ± SD. one-way ANOVA were used to compare the differences. (**p* < 0.05, ***p* < 0.01, ****p* < 0.001. ns indicates no *P*-value meaning)
