# Supplementary material for: MTCH2 regulates NRF2-mediated RRM1 expression to promote melanoma proliferation and dacarbazine insensitivity
Source: Cell Death Dis. 2025 Apr 9;16(1):268. doi: 10.1038/s41419-025-07618-9 (PMC11982210; doi:10.1038/s41419-025-07618-9)

WB original image

Figure 1D

MTCH2：

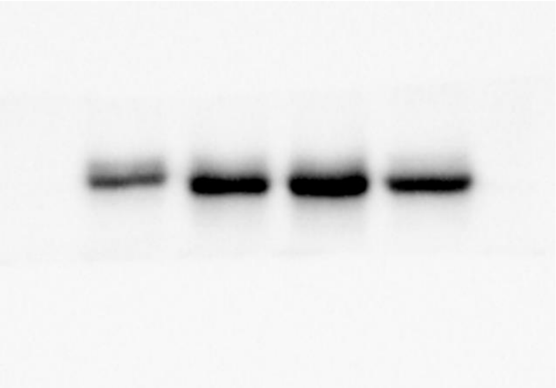

β-actin：

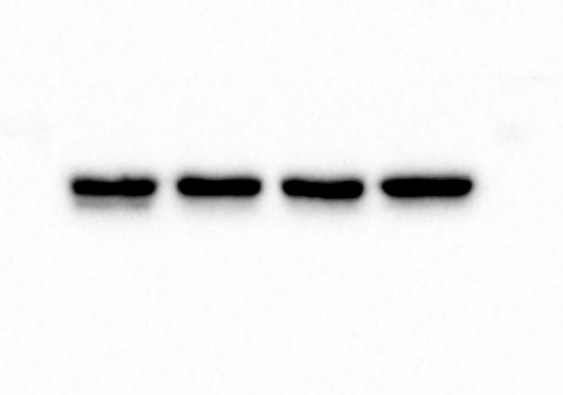

Figure 1G

MTCH2：

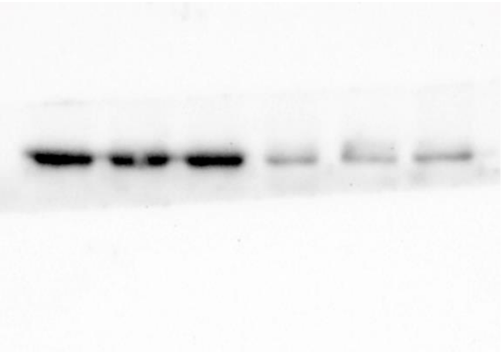

β-actin：

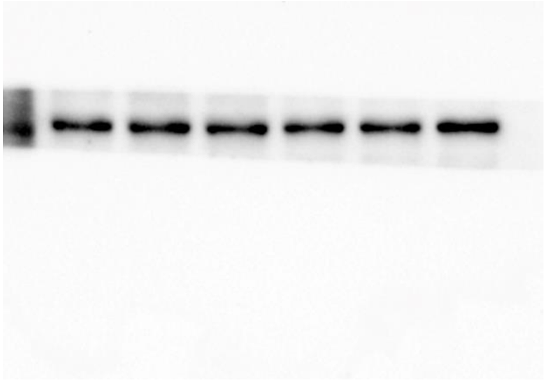

MTCH2：

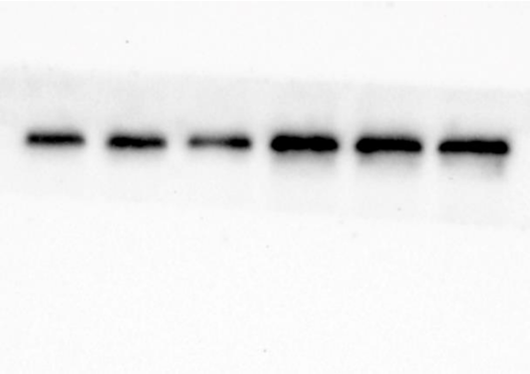

β-actin：

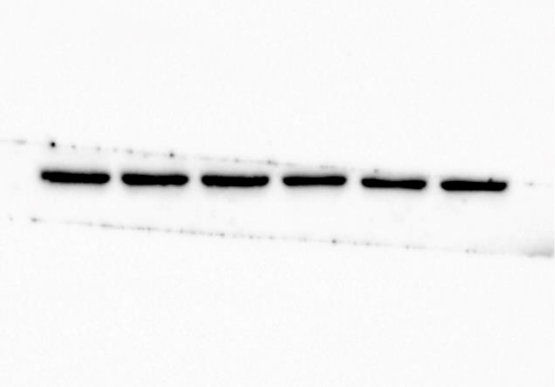

Figure 1H

MTCH2：

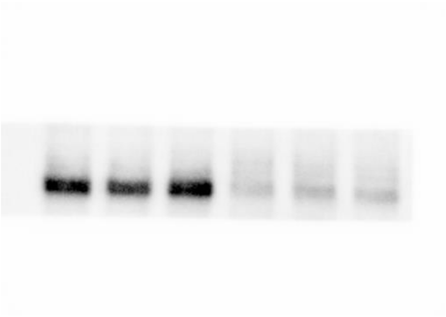

β-actin：

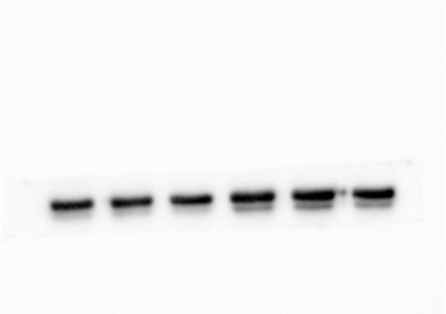

MTCH2：

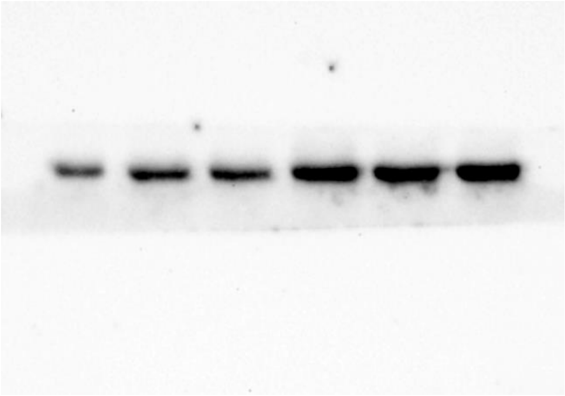

β-actin：

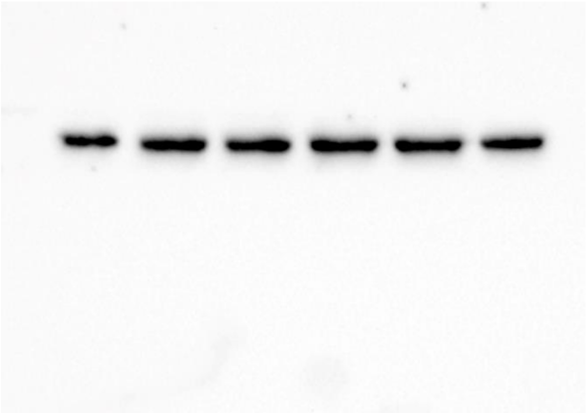

Figure 2E

CyclinE1：

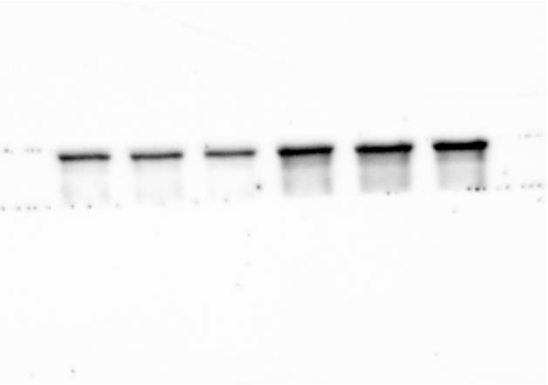

CDK2:

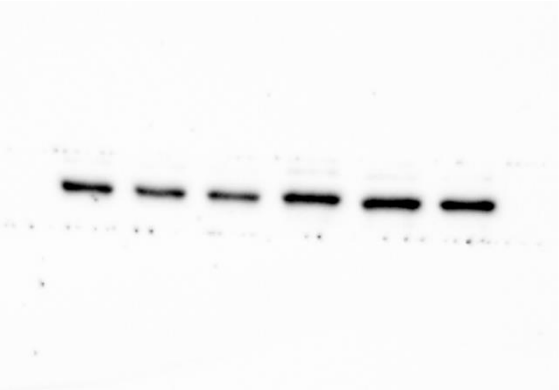

β-actin

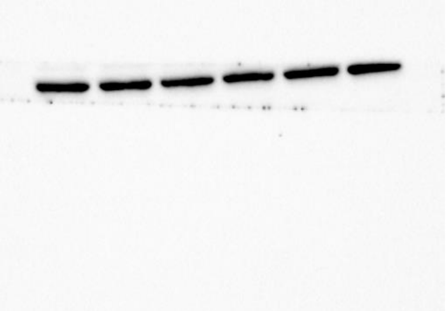

CyclinE1：

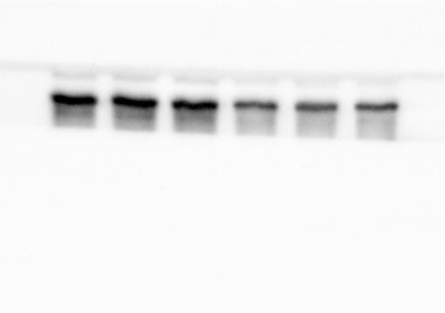

CDK2:

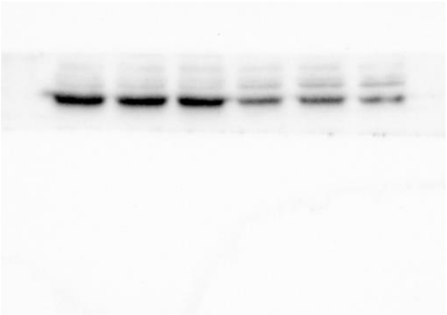

β-actin

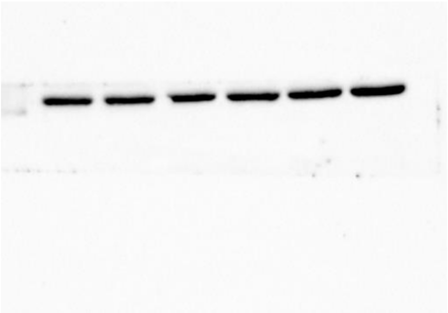

CyclinE1：

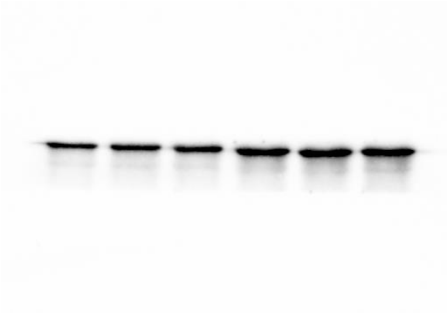

CDK2:

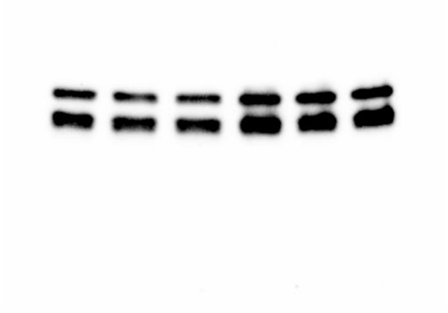

β-actin

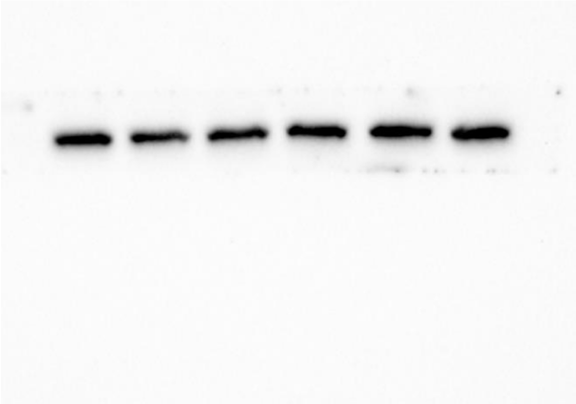

CyclinE1：

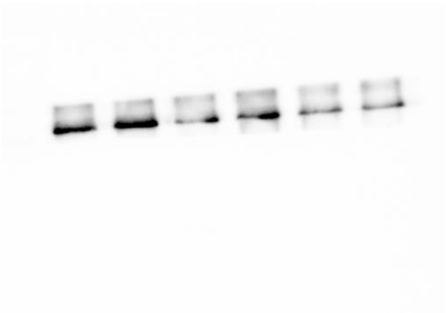

CDK2:

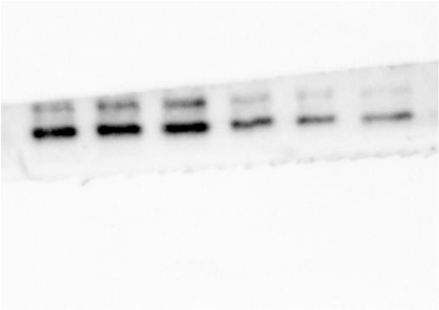

β-actin

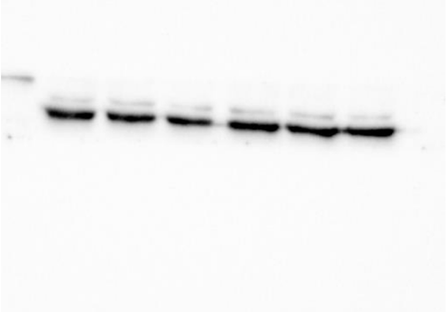

Figure 3C

Bcl2

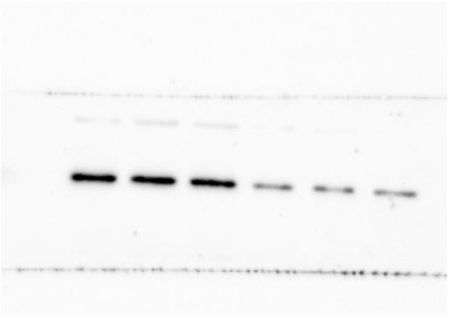

BAX

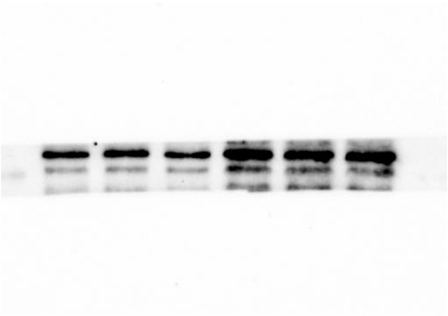

Cl-caspase3

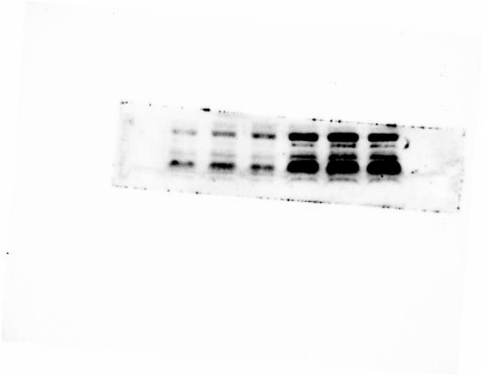

β-actin

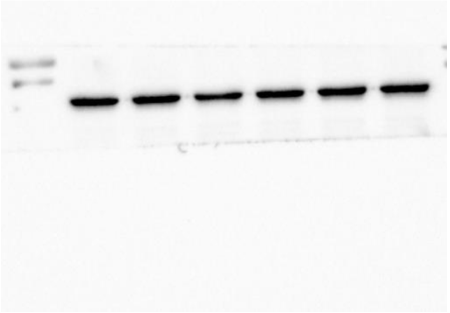

Bcl2

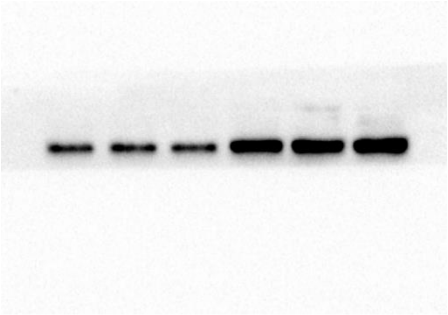

BAX

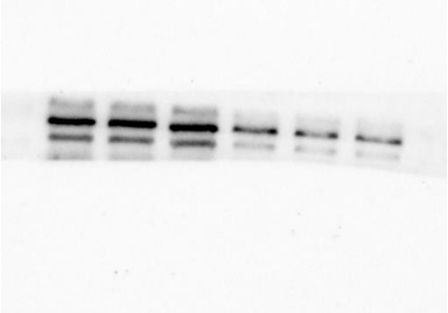

Cl-caspase3

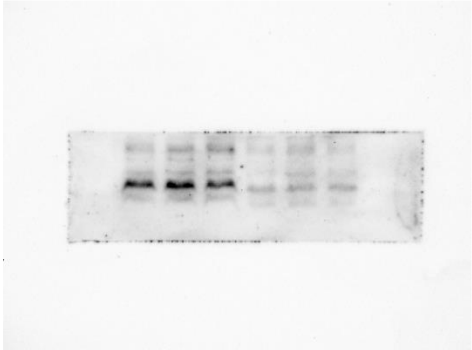

β-actin

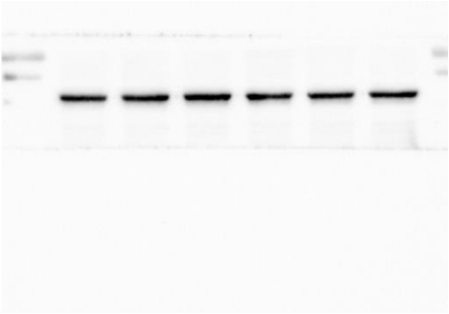

Bcl2

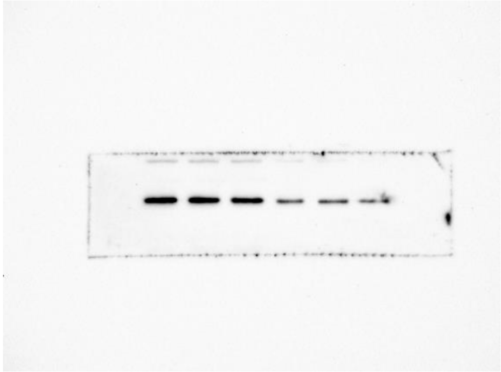

BAX

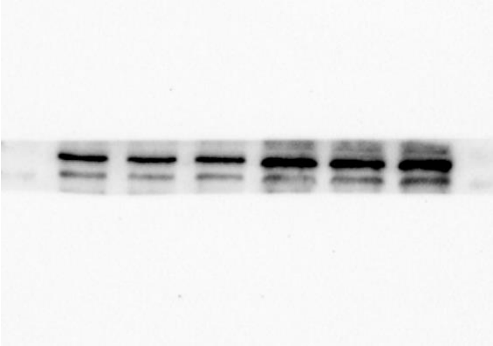

Cl-caspase3

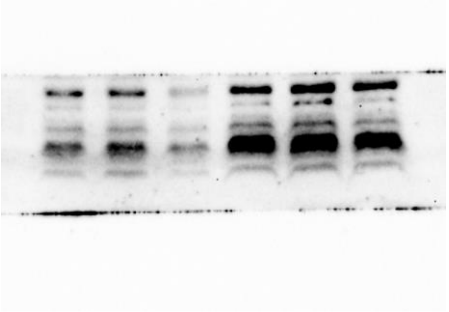

β-actin

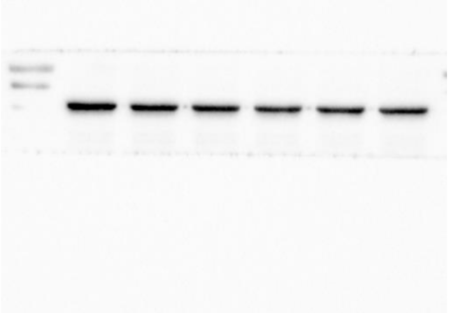

Bcl2

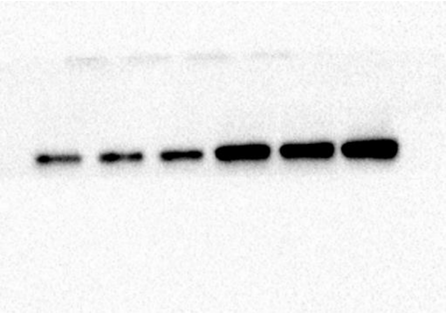

BAX

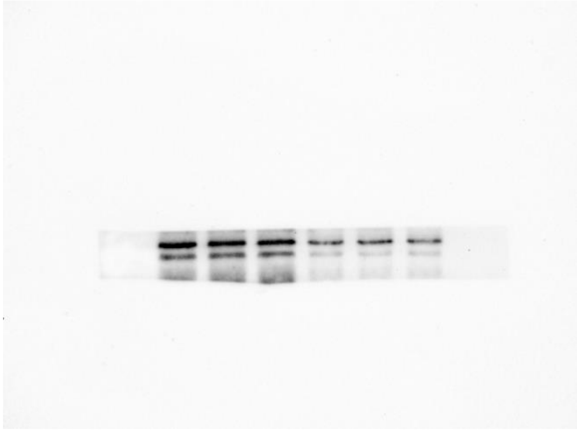

Cl-caspase3

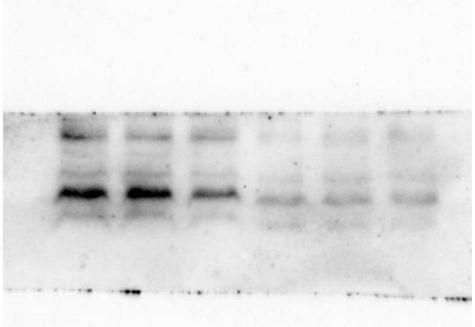

β-actin

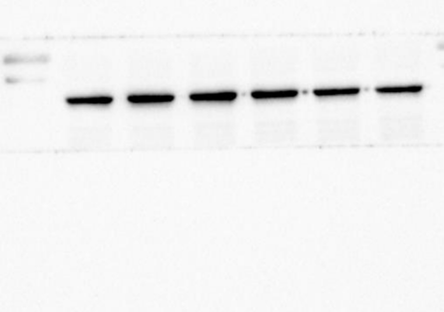

Figure 4C

RRM1

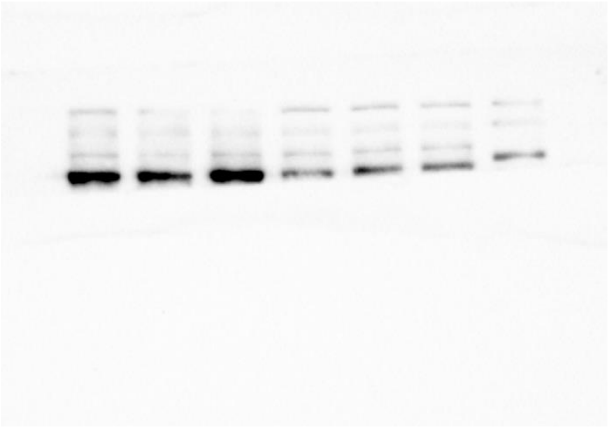

NRF2

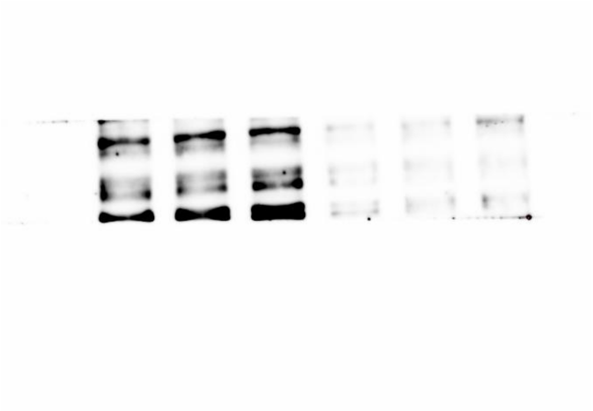

β-actin

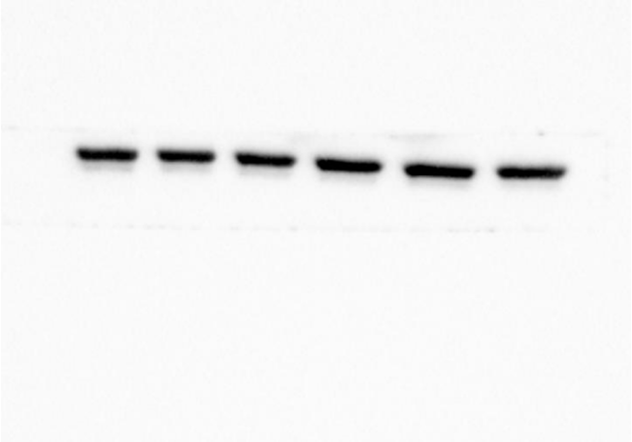

RRM1

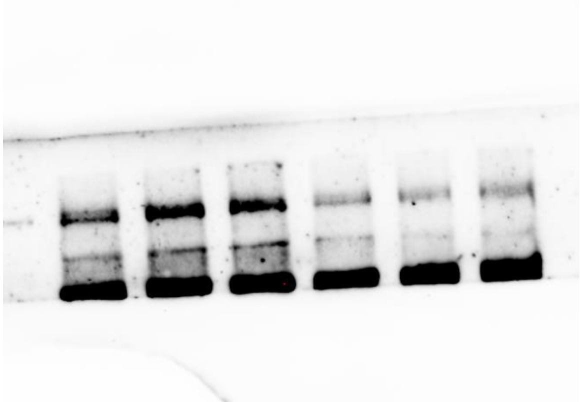

NRF2

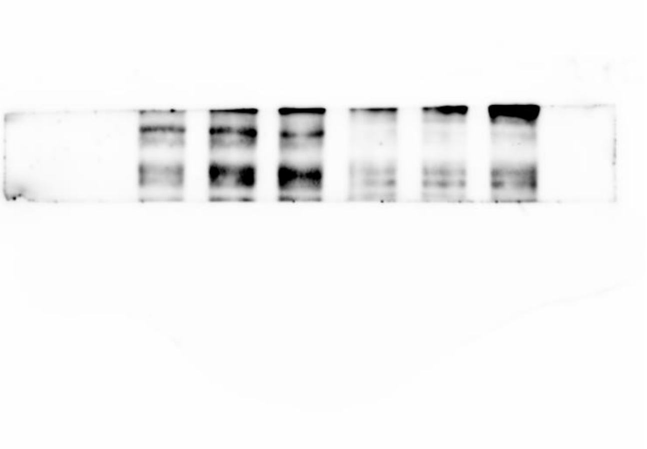

β-actin

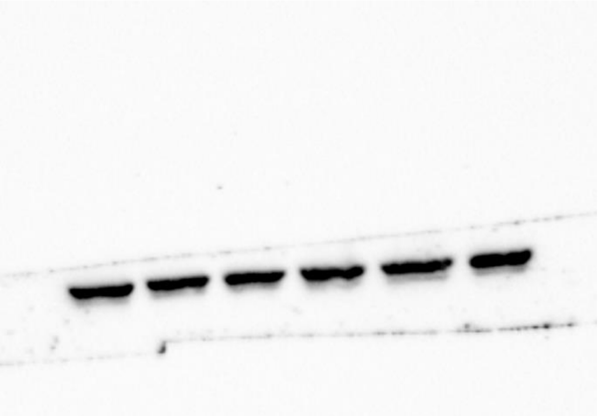

Figure 4D

RRM1

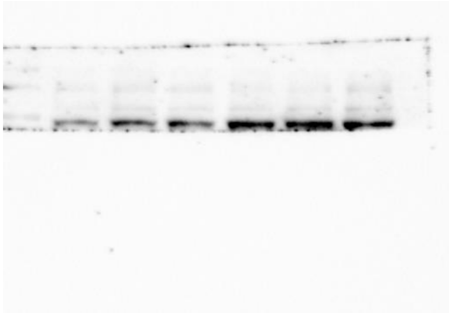

NRF2

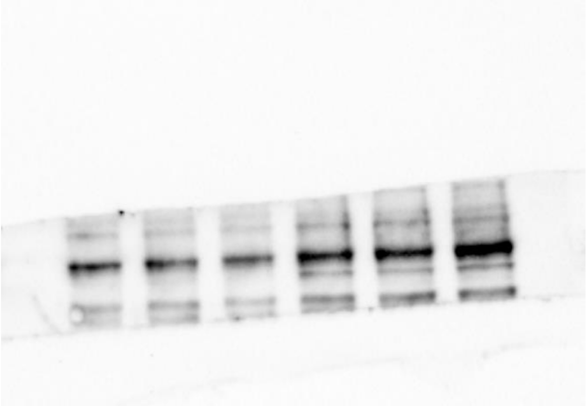

β-actin

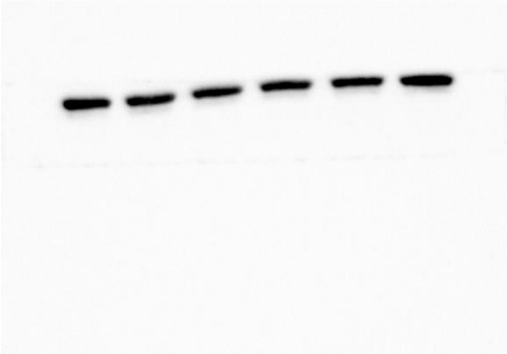

RRM1

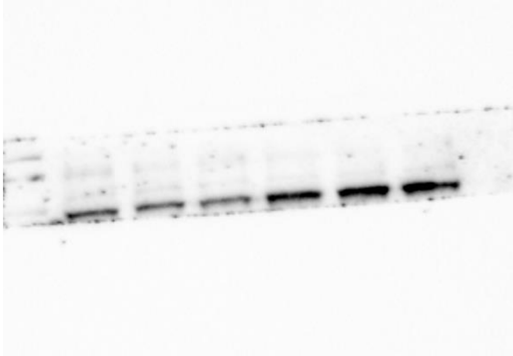

NRF2

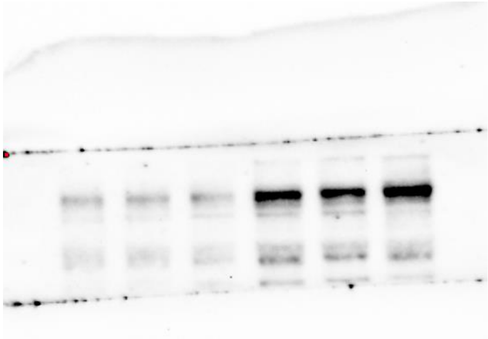

β-actin

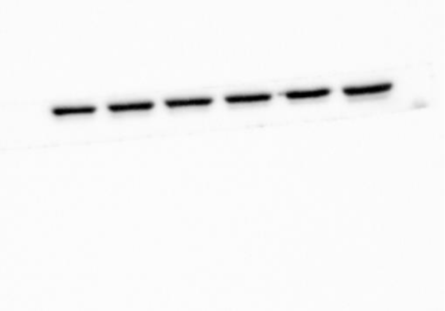

Figure 4F

NRF2

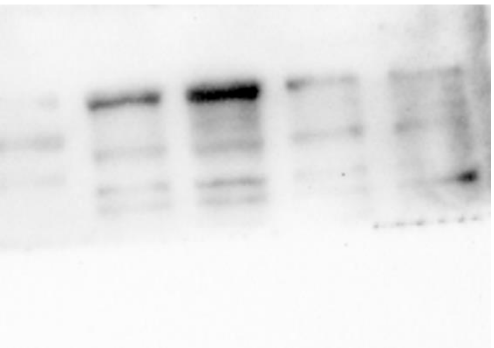

H3

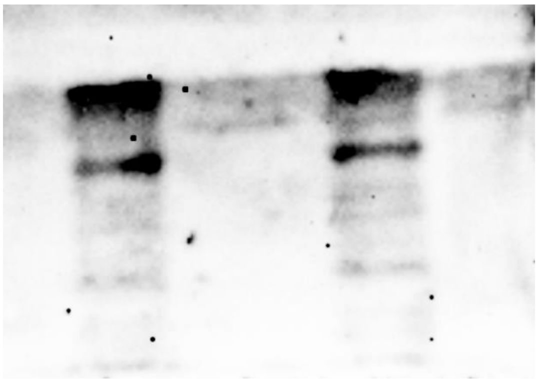

Tubulin

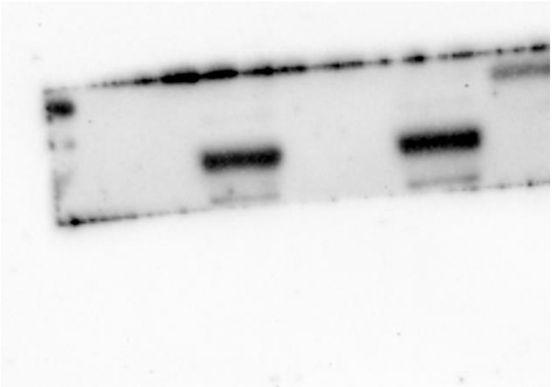

NRF2

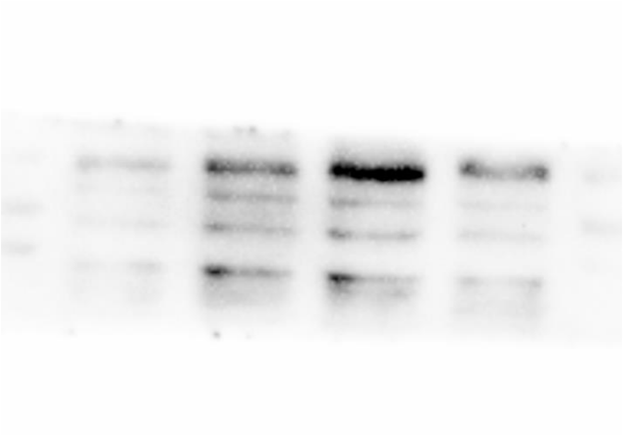

H3

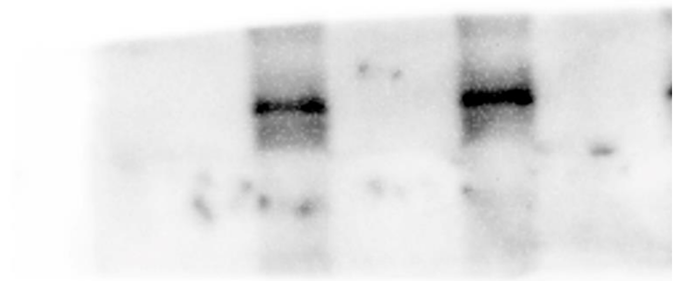

Tublin

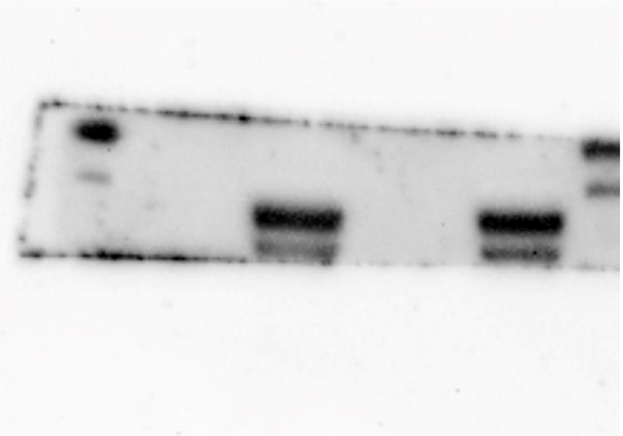

Figure 4G

NRF2

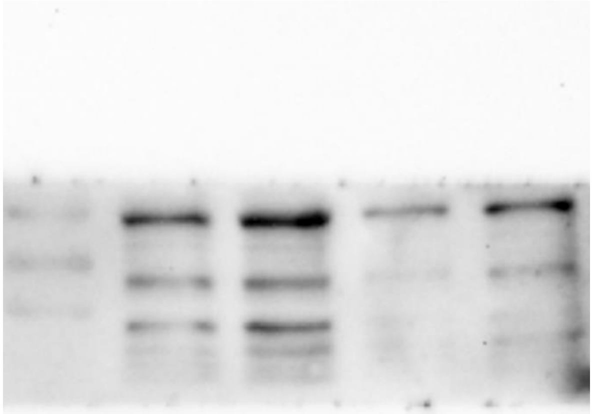

H3

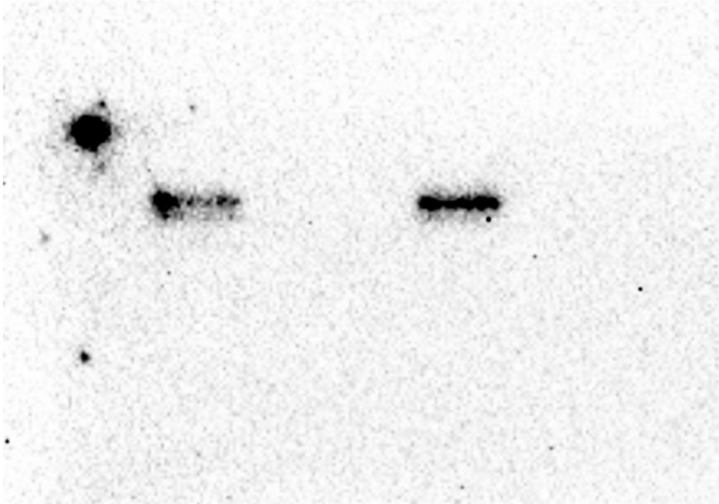

Tubulin

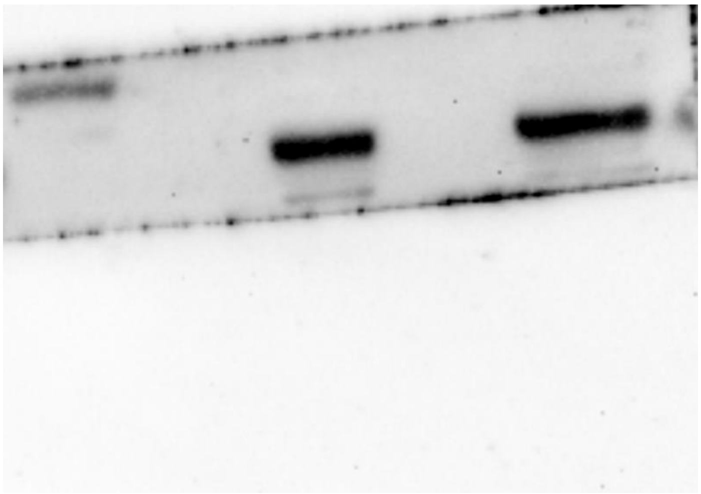

NRF2

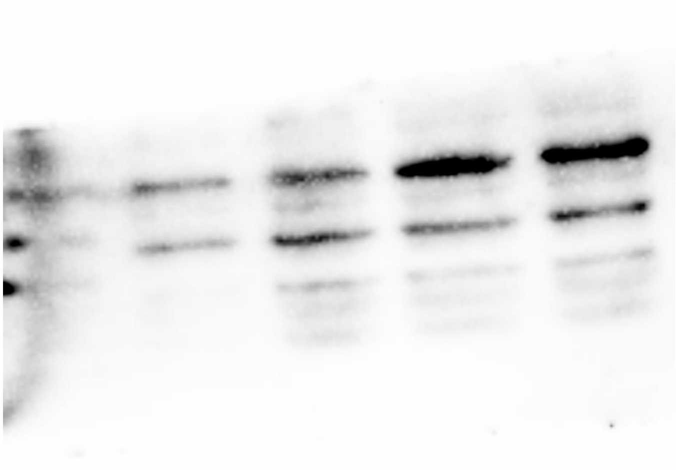

H3

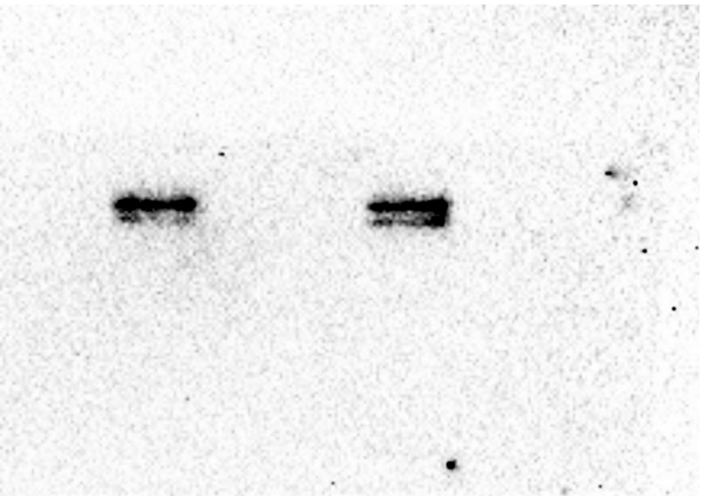

β-actin

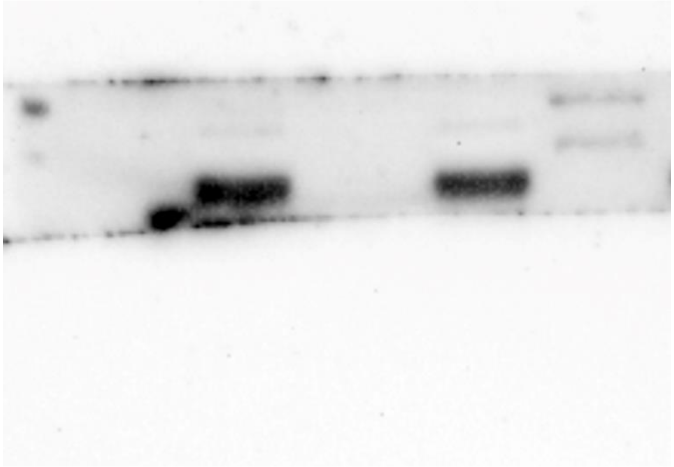

Figure 5A

RRM1

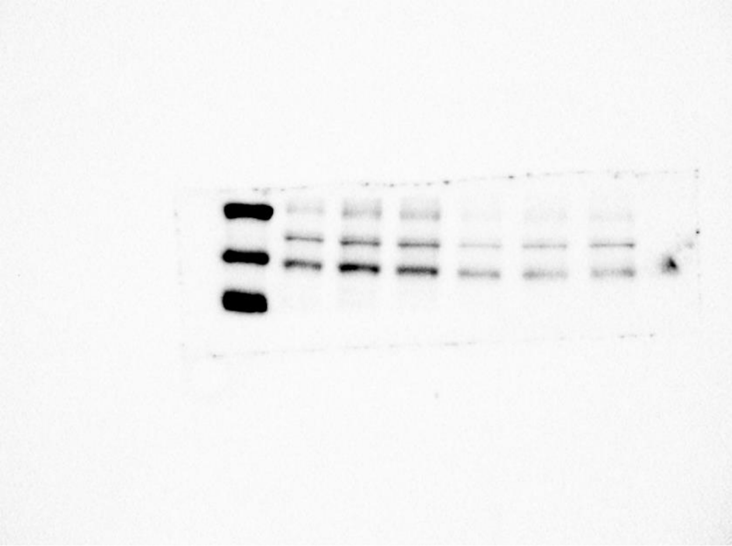

β-actin

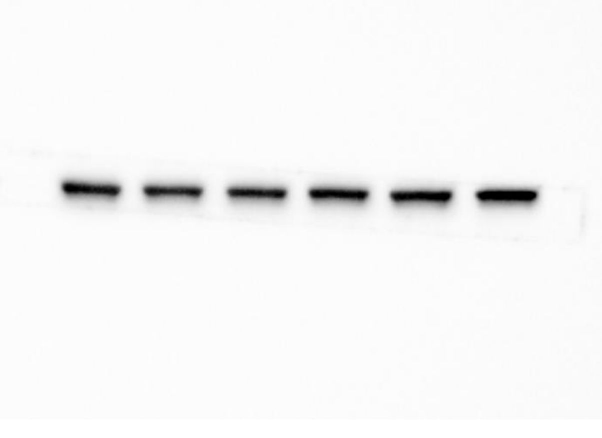

RRM1

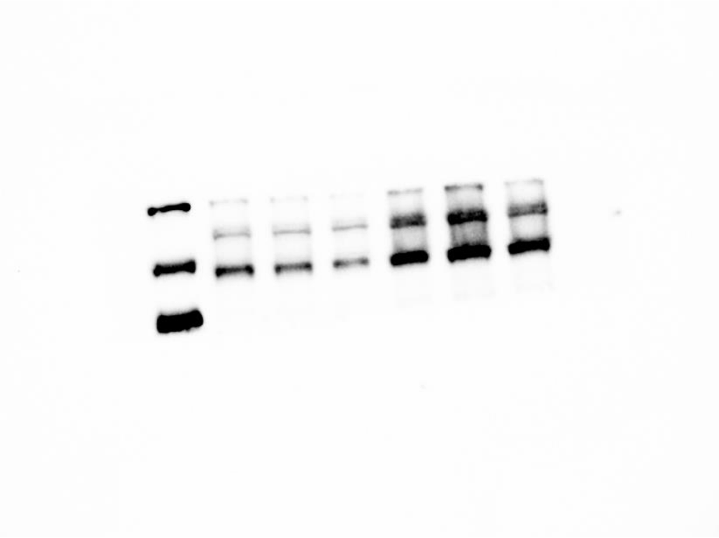

β-actin

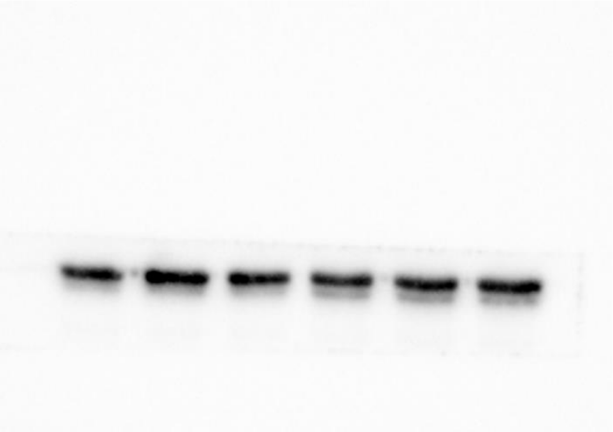

Figure 5B

RRM1

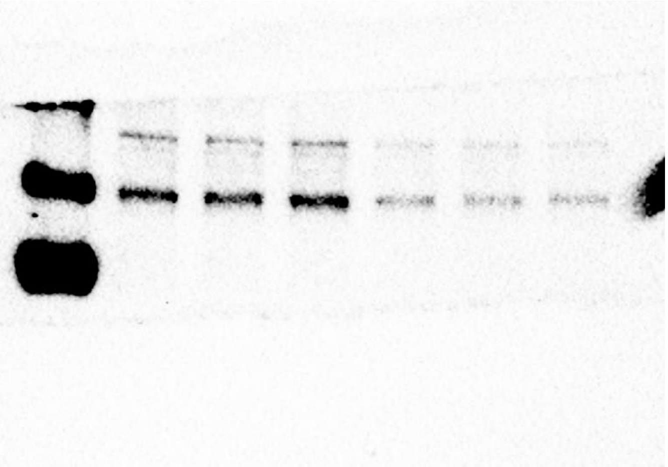

β-actin

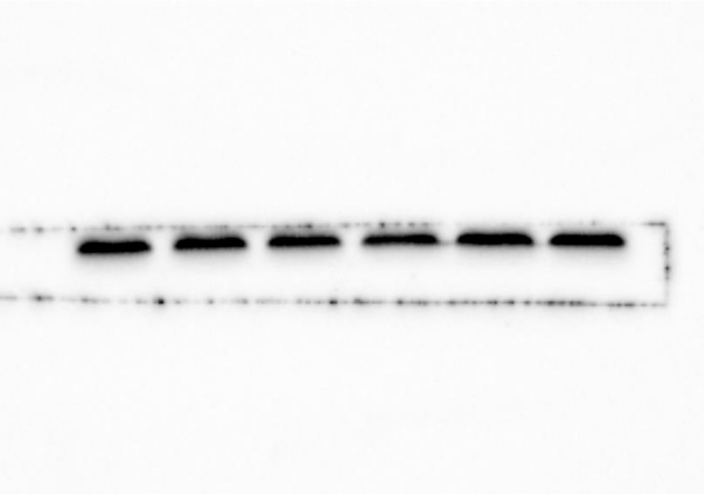

RRM1

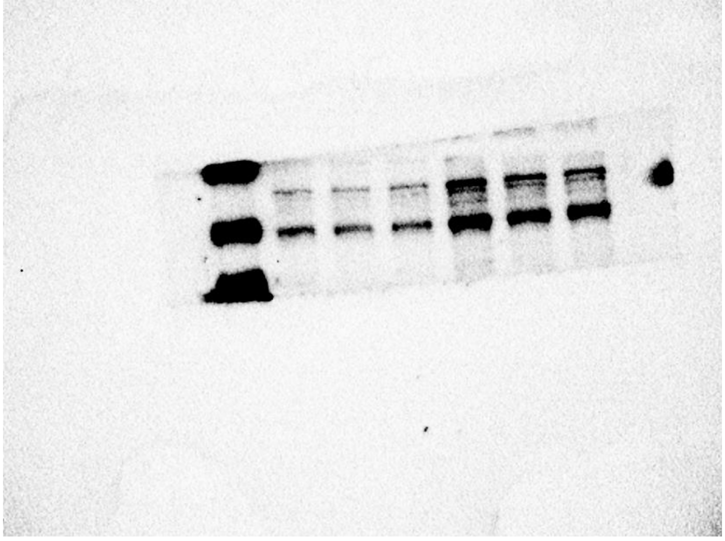

β-actin

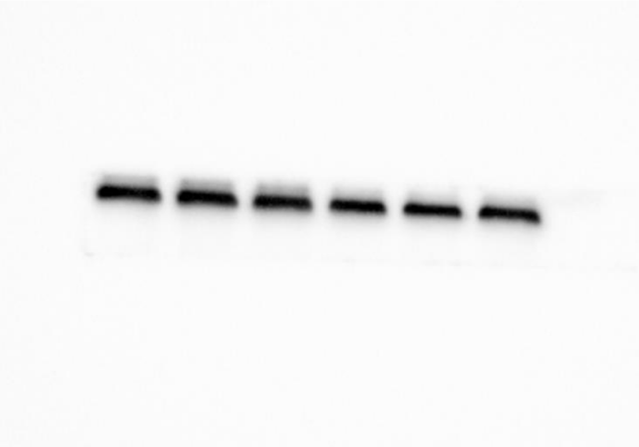

Figure 5E

RRM1

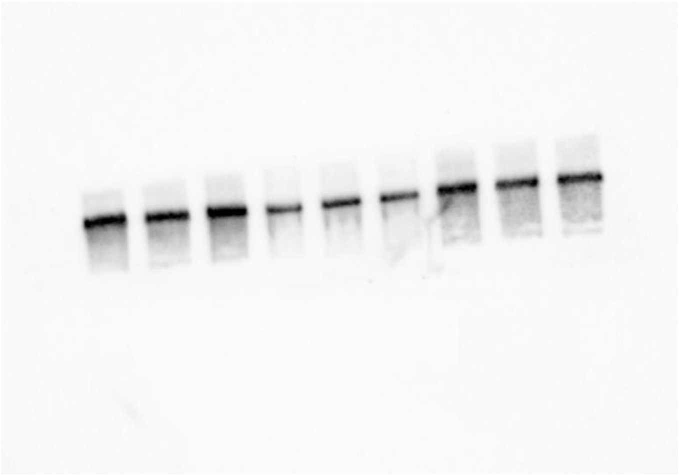

β-actin

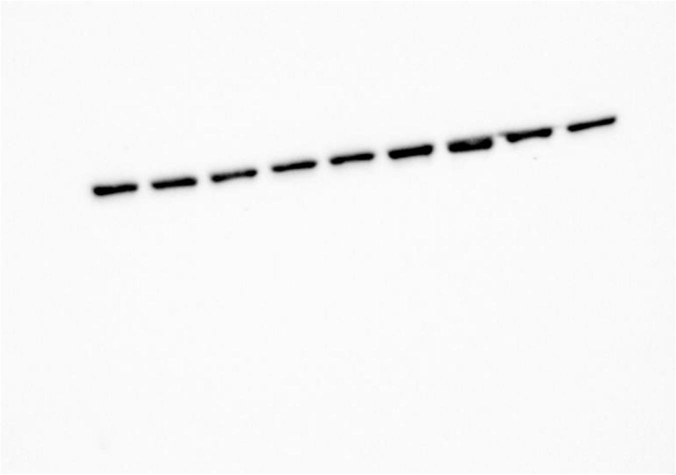

RRM1

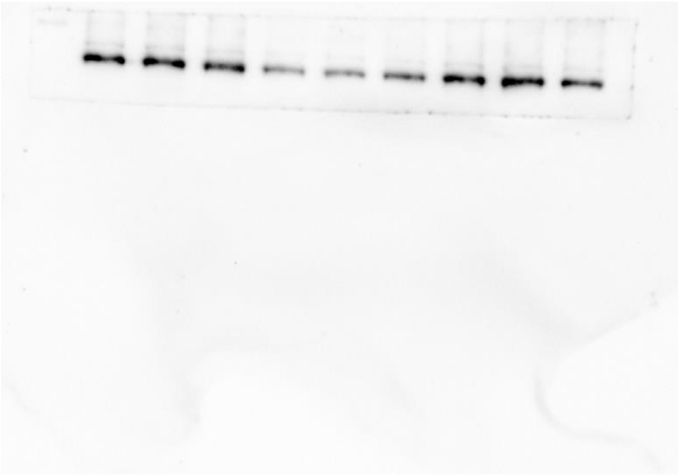

β-actin

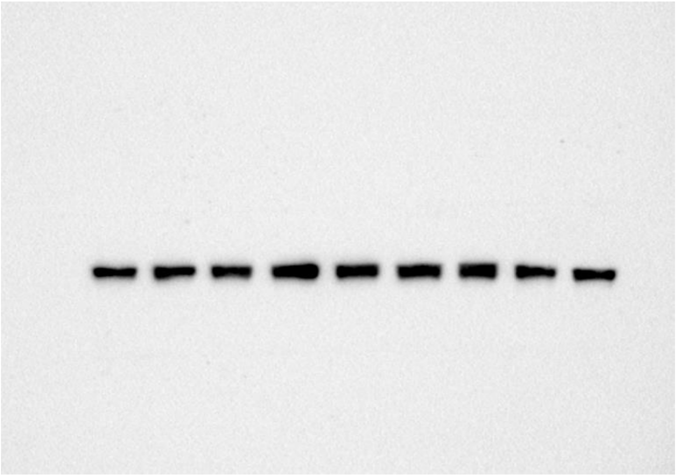

Supplementary Figure 1A

MTCH2

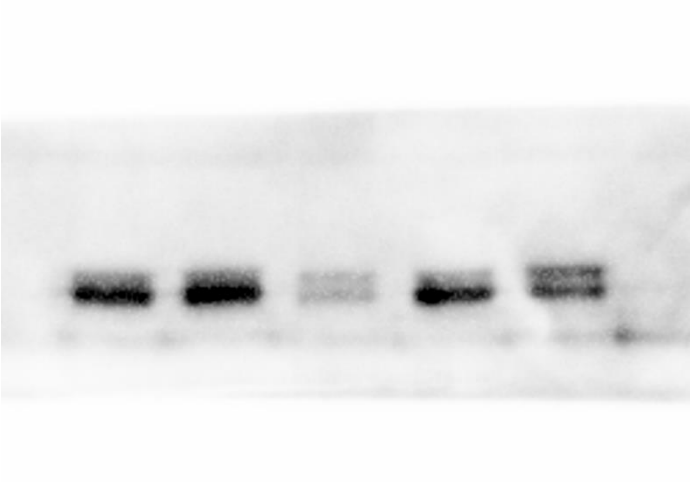

β-actin

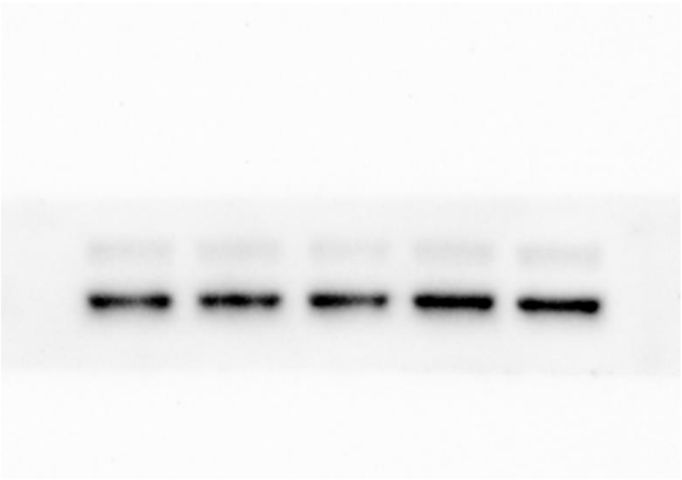

Supplementary Figure 1B

MTCH2

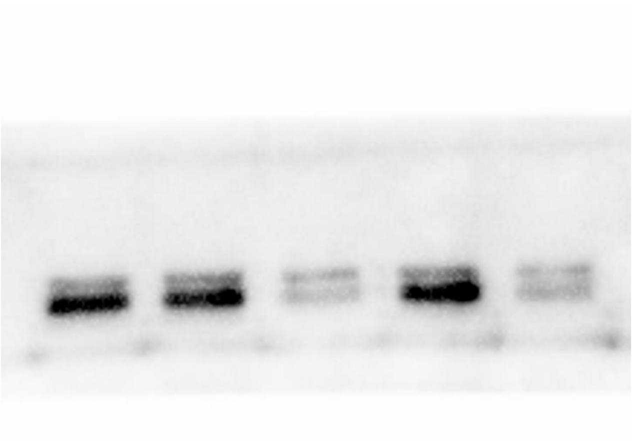

β-actin

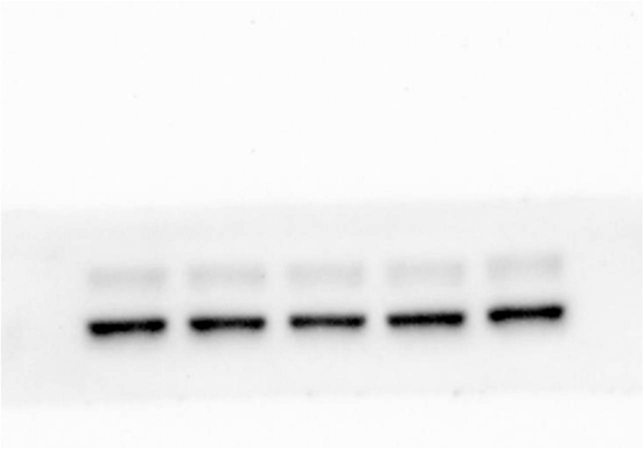

Supplementary Figure 2A

RRM1

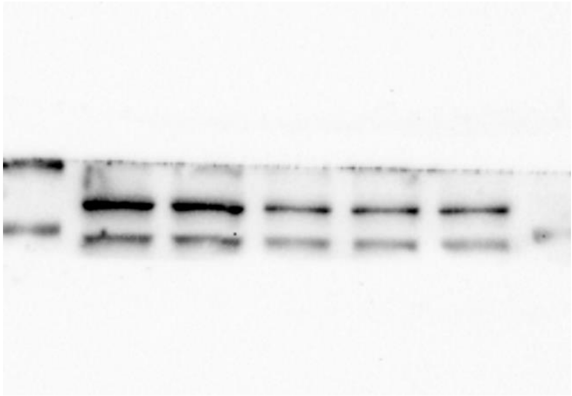

β-actin

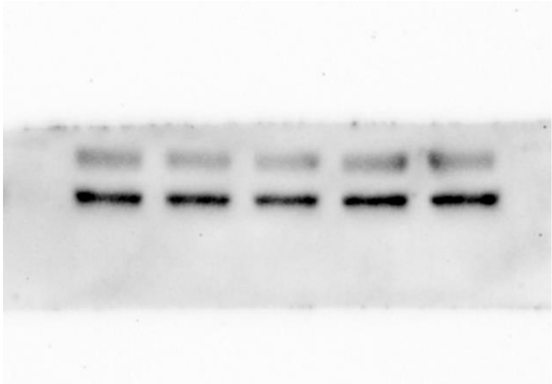

RRM1

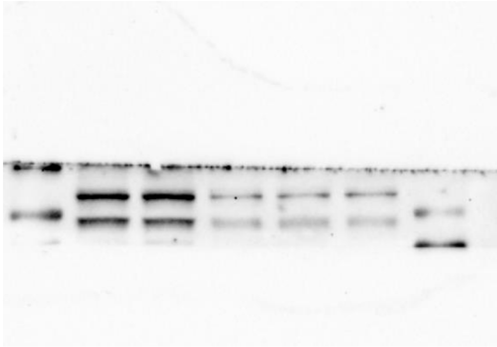

β-actin

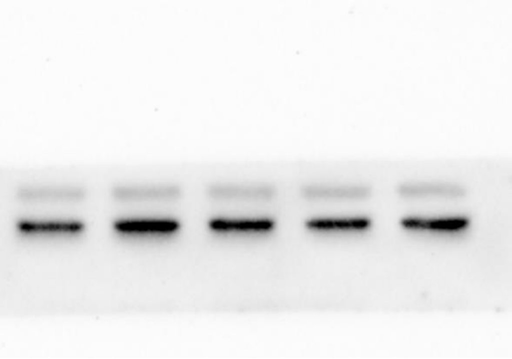

Supplement: Supplementary file 2 — WB original figure [file 41419_2025_7618_MOESM2_ESM.pdf]
